# From cup to cell: phytochemical characterization and safety profiles of some herbal teas in Türkiye

**DOI:** 10.3389/fphar.2026.1803892

**Published:** 2026-04-28

**Authors:** Emre Bayraktaroğlu, Ayfer Beceren, Sevde Nur Biltekin Kaleli, Turgut Taşkın, Erkan Rayaman, İsmail Şenkardeş, Gülden Zehra Omurtag

**Affiliations:** 1 Department of Nutrition and Dietetics, School of Health Sciences, Istanbul Medipol University, Istanbul, Türkiye; 2 Department of Pharmaceutical Toxicology, Faculty of Pharmacy, Marmara University, Istanbul, Türkiye; 3 Department of Pharmaceutical Microbiology, School of Pharmacy, Istanbul Medipol University, Istanbul, Türkiye; 4 Department of Pharmacognosy, Faculty of Pharmacy, Marmara University, Istanbul, Türkiye; 5 Department of Pharmaceutical Microbiology, Faculty of Pharmacy, Marmara University, Istanbul, Türkiye; 6 Department of Pharmaceutical Botany, Faculty of Pharmacy, Marmara University, Istanbul, Türkiye; 7 Department of Pharmaceutical Toxicology, School of Pharmacy, Istanbul Medipol University, Istanbul, Türkiye

**Keywords:** antimicrobial, antioxidant, cytotoxicity, genotoxicity, herbal teas

## Abstract

**Introduction:**

This study was carried out to investigate the antioxidant capacity, antimicrobial, cytotoxicity, and genotoxicity effects of some herbal teas (HTs) grown in Türkiye and sold in pharmacies with medical indications.

**Methods:**

The preparations, prepared to reflect daily usage conditions, were analyzed using the CUPRAC, DPPH, FRAP, and total phenolic content (TPM) assays, the agar well diffusion method, microdilution test, MTT, micronucleus (MN), and comet assays.

**Results:**

FRAP values ranged between 0.956 ± 0.036 and 7.738 ± 0.427 mM FeSO_4_/mg, while the CUPRAC values were 0.14 ± 0.027–5.306 ± 0.107 mM trolox equivalent/mg. Regarding the DPPH assay, the samples exhibited a range of free radical scavenging potencies. TPC values was 0.002 ± 0.00–0.398 ± 0.049 mg gallic acid equivalent/mg. The inhibition zone diameters were 0–10.07 ± 0.55 mm, and the minimum inhibitory concentration values ranged from 0.31 to 0.63 mg/mL. Cytotoxicity tests showed that the IC_50_ values were between 99.68 ± 0.99 μg/mL and 562.45 ± 1.88 μg/mL for A549 cells, 84.02 ± 2.05 μg/mL and 640.22 ± 2.99 μg/mL for MCF7 cells, and 103.99 ± 1.11 μg/mL and 644.55 ± 2.56 μg/mL for HEK293 cells. In the comet assay, DNAT (%) ranged from 28.54 ± 4.49 to 86.88 ± 7.04 µm, while MN frequencies varied between 0% and 11.05% ± 0.13%.

**Discussion:**

Overall, the findings indicate that some herbal teas in the study, such as *Melissa officinalis* and *Origanum vulgare*, exhibit significant antioxidant capacity and anticancer potential along with relatively low genotoxic risk; however, the study also revealed that some herbal teas included in the study may show limited efficacy or potential safety concerns, and it emphasizes the necessity of accurate botanical identification, standardized preparation methods, and comprehensive scientific evaluations prior to their widespread use. Based on the study’s results, it is crucial to establish more extensive public health policies concerning the sale of medicinal herbal teas, emphasizing the standardization of botanical identification and stringent safety certifications to safeguard consumers from potential hazards.

## 1 Introduction

Medicinal herbal teas (HTs) have been one of the frequently used methods for preventing non-communicable diseases for many years, and they are also used in the treatment and prevention of known infectious diseases (Hughes et al., 2015; Mousa, 2017). It is believed that the effect of HTs against diseases is related to the antioxidant capacities of the metabolites they contain (Sugahara et al., 2018; Tang et al., 2013; Zhang et al., 2015). Infectious diseases are illnesses caused by pathogenic microorganisms such as bacteria, viruses, parasites, or fungi, and they can be pose significant challenges to global health and longevity (WHO, 2025). Although the primary treatment method for infectious diseases is antimicrobial drugs, due to the increasing number of diseases, antibiotic resistance, difficulties in accessing modern healthcare services, and high costs, medicinal herbal teas hold significant importance in terms of their antimicrobial efficacy (Hacioglu and Dosler, 2017; Tshivhandekano et al., 2014). Despite the proven positive effects of medicinal herbal teas on health, side effects such as cytotoxicity and genotoxicity, which arise from the bioactive molecules contained in the plants, interactions when used together, and misuse, have also been observed (Abudayyak et al., 2015; Basaran et al., 1996; Bedrood et al., 2018; Najafian et al., 2014). Cytotoxicity studies in healthy cells are very important for evaluating mutagenesis, apoptosis, cellular pathology, and pharmaceutical toxicity. Additionally, the study of cytotoxicity in cancer cells is important from a therapeutic perspective (Komissarova et al., 2005). Genotoxic substances can cause adverse outcomes such as the development and/or predisposition to diseases, increased morbidity/mortality, changes in hereditary traits, and impaired reproductive capacity (Sponchiado et al., 2016). However, it is also known that some substances can be protective against genotoxic effects (Romero-JiménezMuñoz-Serrano and Alonso-Moraga Á, 2005).

This study was conducted to investigate the possible health effects of some HTs grown in Türkiye and sold in pharmacies, with medical indications, on antioxidant, antimicrobial activities, cytotoxicity, and genotoxicity, using methods such as copper (II) ion reducing antioxidant capacity (CUPRAC), 2,2-diphenyl-1-picrylhydrazyl radical scavenging activity determination (DPPH), ferric reducing antioxidant power determination (FRAP), total phenolic content determination (TPC), agar-well diffusion (AWDM) method, minimum inhibitory concentration determination for bacteria (MIC), MTT, micronucleus assay (MN), and alkaline comet assay (ACA). While the phytochemical profiles of these plants are extensively described, the majority of current investigations concentrate on alcoholic extracts or isolated chemicals. This study is unique since it particularly assesses herbal teas available in pharmacies with medical indications, prepared only according to conventional infusion procedures (daily usage) to offer an accurate safety and efficacy profile for consumers.

## 2 Materials and methods

The chemicals used and their preparation are presented in the Supplementary Material.

This study was conducted with ethical approval from İstanbul Medipol University Non-Interventional Clinical Research Ethics Committee (Approval date: 14 September 2022; Approval no: E-108400098-772.02-5291).

### 2.1 Samples and preparation of sample extract

This study included HTs that are sold in pharmacies (to ensure source quality and botanical reliability) in Istanbul, Türkiye, with a medical indication and a specific preparation method, retain the integrity of the plant’s crude drug form (i.e., not in tea bags), are cultivated as part of agricultural production in Türkiye; and are sourced from a single origin. In order to find and get therapeutic HTs that satisfy these criteria, in-person interviews were undertaken with pharmacies situated in different districts of Istanbul. Researchers compiled a list of pharmacies in Istanbul obtained from the Istanbul Chamber of Pharmacists and conducted interviews by randomly visiting the regional representative office and selected pharmacies.

Consequently, 56 mono-herbal and 4 poly-herbal therapeutic HTs were selected and procured directly from a producer in Konya, Türkiye. A plant was omitted from the study due to its unsuitability for use as tea, although being marketed as such. The botanical identification of the plant materials was conducted by Dr. İsmail ŞENKARDEŞ, a faculty member in the Department of Pharmaceutical Botany at the Faculty of Pharmacy, Marmara University. These samples were primarily identified using “Flora of Turkey and the East Aegean Islands” (Townsend and Davis, 1973), and the faculty herbarium, and voucher specimens now deposited in the Marmara University Faculty of Pharmacy Herbarium (MARE), (Voucher No: EB-1-60). The scientific names of taxa were checked using “Turkey Plant List” (A. Güner S. Turkey plant list Vascular plants, 2012) and updated according to World Flora Online (WFO, 2025).

The Turkish and scientific names on the packaging of the HTs examined in the study, along with the names established by botanical identification, are provided in Supplementary Table 1. The study omitted 16 botanical drugs that were either unidentified or identified solely at the genus level.

In order to reflect daily use, each HT was prepared by infusion (60 °C–80 °C) or maceration (25 °C) methods according to the description on each herb at the time of sale. All plants, with the exception of sample 13, sample 14, sample 53, were treated to the infusion procedure. Some botanical drugs were lightly pounded in a marble mortar and pestle before infusion. Detailed descriptions are given in Supplementary Table 2.

The lyophilisation procedures were carried out with Teknosem brand Toros TRS 2/2V model lyophiliser device in the laboratory of İstanbul Medipol University Faculty of Pharmacy, Department of Pharmaceutical Technology. The device was operated after being set to −55 °C, when the device reached −40 °C, the products were placed and the vacuum was opened and adjusted to 10-2 ATM pressure. The samples were kept in the device for 48 h and dried.

Samples 28 and 46 were removed removed before statistical and other analysis from the study due to inadequate dry matter following lyophilization. Half of the lyophilised extracts were dissolved in Dimethyl Sulfoxide (DMSO) at a concentration of 5 mg/mL and transferred to 15 mL centrifuge tubes and the other half were dissolved in LymphoPrime 2 (Capricorn Scientific) at a concentration of 5 mg/mL and stored at −20 °C.

### 2.2 Phytochemical profiling and antioxidant capacity

All phytochemical profiling and antioxidant capacity experiments were repeated three times, and the results were obtained by taking the averages.

#### 2.2.1 Cuprac

Stock solutions of the samples were diluted in methanol to 0.5 mg/mL. For each sample, 60 µL each of the prepared solutions (CuCl_2_ (10 mM), Neocuproine (7.5 mM), Ammonium acetate (1 M)) and extracts and 10 µL of distilled water were added to the wells of the 96-well microplate. For the control, the same amount of distilled water was used instead of the test substance. The covered microplates were kept at room temperature for 1 h. At the end of the time, absorbance values were measured against the control at 450 nm in a microplate reader (Deepa et al., 2013). The Trolox solution was diluted with ethanol at different concentrations, the CUPRAC method was applied, and a calibration equation was obtained by using reference values against the control. The absorbance value of the samples was evaluated by means of the Trolox Equivalent (TE) calibration curve, and the results were expressed as mM TE/mg.

#### 2.2.2 Determination of DPPH radical scavenging assay

Stock solutions of the sample extracts were diluted in methanol to obtain test substances at concentrations of 3 g/mL, 2 g/mL, 1 g/mL and 0.5 g/mL.

For each HT, 240 µL of DPPH solution and 10 µL of the prepared test substances were added to separate microplate wells. The mixture was mixed with a vortex device for 1 min. Then it was kept out of light for 30 min. Ascorbic acid and Butylated Hydroxytoluene were prepared daily under the same conditions as the test substances to be used as standard and the same amount of methanol to be used as control. The absorbance values were measured at 517 nm in a microplate reader at the conclusion of the time (Taşkın and Taşkın, 2018). After all experiments were repeated three times, absorbance values were averaged and DPPH radical scavenging capacity was calculated by the following formula.

$$“\%\text{ DPPH}=\left(\left(C-T\right)\;/\;C\right)\times 100”$$

C=Absorbance value of the control,T=Absorbance value of the test substance

A linear equation was formulated based on the concentrations and DPPH radical scavenging capacity (%) of the standards and samples. The concentration and % scavenging values of each sample were inserted into the linear equation, and the mean SC_50_ (scavenging concentration %50) values of the samples were calculated and expressed in mg/mL.

#### 2.2.3 FRAP

The sample’s stock solutions were diluted in methanol to a concentration of 0.5 mg/mL 190 μL of the produced FRAP reagent was dispensed into each well of a 96-well microplate. Ten microliters of stock solutions were individually applied to each well and incubated for 4 minutes. Ten microliters of DMSO were utilized as a negative control (blind) in three wells. Absorbance measurements at 593 nm were recorded against the blank using a microplate reader. The results were expressed by diluting 1 mM FeSO_4_7H_2_O with distilled water to concentrations of 500, 250, 125, and 62.5 µM, and the experiment was conducted with these solutions. A calibration graph was constructed using the absorbance values measured at 593 nm in relation to the blank, and a regression equation was derived. The absorbance values of the samples were analyzed using the equation, and the findings were presented as mM FeSO_4_/mL (Benzie and Strain, 1996).

#### 2.2.4 TPC determination

Stock solutions of the botanical drugs were diluted in methanol to 0.5 mg/mL. After placing 0.25 µL of diluted extracts and 100 µL of Folin-Ciocalteu reagent into the wells, the mixture was mixed with 75 µL of NaCO_3_ after 4 min. It was kept in a shaking water bath at room temperature for 2 h. At the end of the period, it was measured at 760 nm against the negative control. The above method was repeated with different concentrations of gallic acid, and a calibration curve was obtained from the changes in absorbance values. The total phenolic content of the test substances was calculated using this curve, expressed as mg Gallic Acid Equivalent (GAE), (Taşkın et al., 2011).

The values derived from the antioxidant activity testing of the HTs were organized in descending order (and conversely for DPPH), and the rank numbers were aggregated. The research proceeded with the botanical drugs in the lowest third percentile. The research proceeded with the 12 botanical drugs exhibiting the most significant antioxidant activity according to the results collected.

### 2.3 Antimicrobial activity

The bacterial strains *Streptococcus mutans* ATCC 25175, *Streptococcus pyogenes* ATCC 19615, *Streptococcus pneumoniae* ATCC 49619, *Staphylococcus aureus* ATCC 29213, *Staphylococcus epidermidis* ATCC 12228, *Escherichia coli* ATCC 25922, *Pseudomonas aeruginosa* ATCC 27853, *Proteus* vulgaris ATCC 13315, *Klebsiella pneumoniae* ATCC 4352, and the yeast strain *Candida* albicans ATCC 90028 were obtained from the collection of the Department of Pharmaceutical Microbiology, Faculty of Pharmacy, Marmara University. S. mutans, S. pyogenes, and S. pneumoniae were cultured in 5% sheep blood Mueller-Hinton Agar at 37 °C for 24 h, other bacterias in Tryptic Soy Agar at 37 °C for 24 h, and C. albicans in Sabouraud Dextrose Agar at 35 °C for 48 h.

#### 2.3.1 Agar-well diffusion method

All microorganisms were cultivated by inoculating appropriate media and incubated at the optimal temperature. Suspensions of microorganisms were generated from colonies on solid media in 0.85% physiological saline (PS). Suspensions of microorganisms were prepared in physiological saline (PS) and adjusted to a turbidity corresponding to the McFarland 0,5 standard for bacteria and the McFarland 1 standard for yeast. Using a sterile swab, the suspensions were applied to the surface of the Mueller-Hinton Agar medium. Wells with a diameter of 5 mm were formed at regular intervals on the culture medium using a sterile Pasteur pipette, into which 50 µL of the samples were added. Petri dishes were incubated at 37 °C for 24 h, following which the zones of inhibition were measured in millimeters and the averages computed. Moreover, meropenem (10 µg/well) for bacteria, amphotericin B (100 µg/well) for yeast, and PS used as controls. The experiments were performed in triplicate (Perez, 1990; Bush and Dudley, 2009).

#### 2.3.2 Determination of minimum inhibitory concentration (MIC) and minimum bactericidal concentration (MBC) for bacteria

MIC determination was performed in accordance with Clinical and Laboratory Standards Institute (CLSI) standards. The bacteria were cultivated under appropriate conditions, and a bacterial suspension was prepared from the colonies in a 24-h bacterial culture according to McFarland 0.5 turbidity, and diluted to achieve a final inoculum concentration of 5 × 10^5^ CFU/mL 100 μL of Cation-adjusted Mueller Hinton (CAMHB) broth was placed in sterile U-bottom microdilution plates, and samples were added to the first wells of the microplate with 100 μL, followed by serial dilutions. Then, 5 µL of bacterial suspension was added to the wells containing the samples, and they were incubated at 37 °C for 24 h. At the end of the incubation, the lowest sample concentration at which no growth was observed was determined as the MIC. CAMHB and meropenem were used as controls. In the determination of the minimum bactericidal concentration (MBC), 5 µL was taken from wells where no growth was observed in the MIC test, added to tryptic soy agar medium, and incubated in Petri dishes at 37 °C for 24 h. After incubation, the lowest concentration without microbial growth was determined as the MBC (Bush and Dudley, 2009; Cockerill et al., 2012).

### 2.4 *In Vitro* cell line assessments

Cytotoxicity assays were conducted using Human Lung Cancer A549 (ATCC CCL-185), Human Breast Cancer MCF7 (ATCC HTB-22), and Healthy Human Embryonic Kidney HEK293 (ATCC CRL-1573) cell lines.

Cell lines sourced from American Type Culture Collection were cultured in Dulbecco’s Modified Eagle’s Medium with High Glucose, enriched with 10% fetal bovine serum and 1% antibiotic, in a 5% CO_2_ atmosphere at 37 °C. Following a 24-h incubation period, their vitality was assessed with an inverted microscope. The culture media was discarded from the dish, followed by rinsing with Phosphate-buffered Saline (PBS), and the cells were detached using 1 mL of Trypsin/EDTA. The cells were allocated to 96-well microplates, with 200 µL each well, comprising 5 X 10^4^ cells per well. Subsequent to incubation, the culture media was extracted. Herbal tea extracts were introduced to the cultured cells at six different concentrations, with 200 µL culture medium serving as a negative control and colchicine as a control followed by a 24-h incubation period. Following incubation, the culture medium were discarded, 30 µL of MTT stock solution was introduced, and after a 4-h period, 150 µL of isopropanol was added. Subsequent to the agitation of the solution, the absorbance was quantified at a wavelength of 570 nm. The absorbance values of the solutions containing the extracts were compared to the negative control (Sahin et al., 2021; Mosmann, 1983).

The IC_50_ value was calculated on a computer. Each experiment was conducted in triplicate. Cell viability was calculated using the following formula:

$$\begin{array}{c} \%\text{ Viability}=\left(Sample\;\left(A570nm\right)-Blank\;\left(A570nm\right)\right)\\ /\;\left(Control\;\left(A570nm\right)-Blank\;\left(A570nm\right)\right)\;X\;100 \end{array}$$

### 2.5 Genotoxicity assessment

Peripheral blood was obtained from two healthy women and two healthy men aged 20–25, who had not taken any medication in the preceding week, refrained from tobacco and alcohol consumption, and had no exposure to medical radiation in the last 6 months, following the acquisition of written informed consent.

In this investigation, the genotoxicity assessments performed with the IC_50_ values obtained from cell trials, half of the IC_50_ values, and double the IC_50_ values of the plant extracts yielded an unquantifiable level of harm; hence, these concentrations were diluted tenfold, and the procedures were repeated.

#### 2.5.1 Alkaline comet assay

One hundred microliters of the obtained blood samples were transferred to a microcentrifuge tube containing nine hundred microliters of PBS, inverted, and thereafter stored in the refrigerator for 10 minutes. 100 μL of Histopaque-1077 was deposited at the base of the microcentrifuge tube and subjected to centrifugation for 5 min at +4 °C and 1060 RPM. The lymphocytes gathered on the upper surface were transferred to a distinct microcentrifuge tube. Each HT extract was introduced into the tubes at the concentrations delineated in Supplementary Table 3, while lymphocytes were added in a microcentrifuge tube for samples, and negative (medium), and positive (H_2_O_2_) controls, incubated at 37 °C for 30 min, and subsequently centrifuged at 300 rpm for 5 min.

Low melting agar and lymphocytes were combined in equal volumes of 100 μL, and the resultant combination was applied to slides coated with high melting agar. The slides were thereafter covered with coverslips and incubated in the refrigerator for 20–25 min. The slide covers removed from the refrigerator were opened and placed in trays containing lysis solution, which were then covered with aluminum foil and stored in the refrigerated for 1 hour. Subsequently, the slides were maintained in an alkaline buffer for 20 min to acclimate them to the alkaline environment. The prepared samples underwent electrophoresis at 25 V/cm and 300 mA for a duration of 20 min. Subsequently, they were immersed in a buffer solution for 5 min to achieve neutralization and prepare for visualization. Upon completion of neutralization, it was progressively rinsed with 50%, 75%, and 100% ethanol for 5 min each (Tice et al., 2000).

Fifty microliters of ethidium bromide was applied to the slides and thereafter covered with coverslips. Fluorescence microscopy was conducted, and the images were analyzed utilizing the Comet Image Processing and Analysis Software System (BAB Bs 200 Pro). The measured DNAT (%) were analyzed using a one-way analysis of variance and Post Hoc Tukey tests.

#### 2.5.2 Micronucleus assay

0.2 mL of the obtained blood samples were cultured in tubes containing 2.5 mL of culture media at 37 °C for a duration of 72 h. At the 24th hour of incubation, extracts derived from HTs were introduced in the quantities detailed in Supplementary Table 4, alongside positive (Mitomycin C) and negative controls. Cytochalasin B was administered at the 44th hour of incubation. Upon completion of the incubation time, the tubes underwent centrifugation at 1,000 rpm for 10 min, after which the supernatants were discarded. Five milliliters of a hypotonic solution (0.075 M KCl) was added to each tube using a vortex mixer. The tubes were refrigerated for 5 min, the centrifugation was repeated, and the supernatant was discarded. A fixative solution of 3 parts methanol and 1 part acetic acid will be added to the tubes in a volume of 5 mL. After a 15-min incubation in the refrigerator, the tubes will be centrifuged again, and the supernatant will be discarded. This procedure will be executed twice. Formaldehyde was incorporated into the fixative solution, aliquoted into 5 mL tubes, centrifuged, and the supernatant was removed. The acquired solutions were distributed on sterile slides and subjected to staining with 5% Giemsa stain. The slides were inspected under a light microscope post-staining, with 1,000 cells evaluated each subject, resulting in a total of 4,000 cells from 4 individuals. The quantities of micronuclei, binucleates, multinucleates, buds, bridges, trinucleates, mononucleates, necrosis, and apoptosis within the 1,000 cells were documented. The Nuclear Division Index (NDI) was computed using data derived from the initial 500 cells. Micronucleus counts were expressed as a percentage. Micronucleus generation based on dosage was evaluated by linear regression analysis (Şen, 2018; Decordier and Kirsch-Volders, 2006; Fenech, 2002; OECD, 2016).

## 3 Results

Following botanical identification, 9 of the 55 mono-herbal HTs initially considered appropriate were determined to be inconsistent with the plant specified on the label. Sixteen samples remain either unidentified or are described just at the genus level. Given that these conflicting mono-HTs were also present in blended teas, four poly-herbal teas were likewise deemed inconsistent with the label. Consequently, it was ascertained that 13 (%22,03) out of 59 HTs were inconsistent with the labeling information.

The outcomes of the TPC, CUPRAC, FRAP, and DPPH radical scavenging capacity studies are presented in Table 1. *Melissa officinalis* exhibited the highest total phenolic content. The CUPRAC experiments revealed that *Melissa officinalis* exhibited the highest value at 5.306 ± 0.107, while *Plantago lanceolata* L. showed the lowest value at 0.14 ± 0.027. FRAP experiments indicated that *Melissa officinalis* exhibited the highest effect (7.738 ± 0.427), while *Morus nigra* L. demonstrated the lowest effect (0.956 ± 0.036). The DPPH radical scavenging capacity assays are quantified using SC_50_ values, where a higher value indicates lower capacity. The highest value was observed in *Melissa officinalis* (0.011 ± 0.002), while the lowest value was recorded in *Plantago lanceolata* L. (0.683 ± 0.034).

**TABLE 1:** TPC, CUPRAC, FRAP, and DPPH radical scavenging assay.

Sample no.	Sample name	CUPRAC	FRAP	DPPH	Total phenolic content
		mM TE/mg extract	mM FeSO_4_/mg extract	SC_50_ (mg/mL)	mg GAE/mg extract
2	*Melissa officinalis* L.	5.306 ± 0.107	7.738 ± 0.427	0.011 ± 0.002	0.398 ± 0.049
3	*Origanum onites* L.	3.161 ± 0.281	4.016 ± 0.16	0.044 ± 0.003	0.087 ± 0.009
4	*Origanum vulgare* subsp. *hirtum* (Link) Ietswaart	4.851 ± 0.487	5.304 ± 0.073	0.02 ± 0.001	0.141 ± 0.014
5	*Sambucus nigra* L.	2.266 ± 0.069	2.922 ± 0.079	0.051 ± 0.002	0.047 ± 0.002
7	*Hypericum perforatum* L.	2.574 ± 0.131	3.283 ± 0.053	0.044 ± 0.006	0.033 ± 0.026
8	*Valeriana officinalis* L.	1.573 ± 0.064	2.69 ± 0.115	0.047 ± 0.002	0.013 ± 0.011
9	*Lavandula* x *intermedia* Emeric ex Loisel.	2.905 ± 0.134	4.309 ± 1.51	0.056 ± 0.002	0.076 ± 0.006
10	*Matricaria chamomilla* var. *recutita* (L.) Fiori	1.087 ± 0.041	2.262 ± 0.009	0.119 ± 0.028	0.002 ± 0.001
11	*Rosa damascena* Mill.	2.44 ± 0.274	3.164 ± 0.056	0.046 ± 0.004	0.077 ± 0.024
12	*Tilia tomentosa* Moench	0.959 ± 0.075	2.186 ± 0.018	0.077 ± 0.008	0.004 ± 0.002
13	*Althaea officinalis* L.	0.669 ± 0.056	2.207 ± 0.005	0.254 ± 0.038	0.015 ± 0.018
16	*Plantago lanceolata* L.	0.14 ± 0.027	1.843 ± 0.038	0.683 ± 0.034	0.011 ± 0.004
17	*Salvia fruticosa* Mill.	2.816 ± 0.147	3.201 ± 0.132	0.045 ± 0.008	0.075 ± 0.005
18	*Salvia officinalis* L.	2.088 ± 0.07	2.887 ± 0.048	0.052 ± 0.004	0.044 ± 0.002
21	*Lycium barbarum* L.	0.54 ± 0.03	1.913 ± 0.172	0.172 ± 0.013	0.021 ± 0.013
22	*Urtica dioica* L.	1.189 ± 0.01	2.402 ± 0.132	0.185 ± 0.011	0.022 ± 0.004
23	*Aronia melanocarpa* (Michx.) Elliott	0.534 ± 0.053	2.137 ± 0.035	0.147 ± 0.02	0.025 ± 0.002
24	*Cichorium intybus* L.	0.79 ± 0.078	2.312 ± 0.124	0.451 ± 0.043	0.016 ± 0.012
26	*Calendula officinalis* L.	0.715 ± 0.101	2.131 ± 0.08	0.174 ± 0.032	0.004 ± 0.002
29	*Vitis viniferae L.*	2.555 ± 0.204	3.065 ± 0.16	0.039 ± 0.008	0.051 ± 0.007
32	*Artemisia absinthium L.*	1.202 ± 0.037	2.338 ± 0.074	0.069 ± 0.012	0.018 ± 0.004
33	*Crataegus azarolus var. aronia L.*	0.367 ± 0.031	1.756 ± 0.092	0.291 ± 0.062	0.017 ± 0.003
37	*Echinacea purpurea* (L.) *Moench*	2.382 ± 0.19	2.585 ± 0.4	0.049 ± 0.003	0.053 ± 0.004
39	*Rubus idaeus* L.	1.37 ± 0.048	2.088 ± 0.015	0.033 ± 0.003	0.04 ± 0.034
40	*Galega officinalis* L.	1.075 ± 0.086	1.864 ± 0.413	0.085 ± 0.015	0.01 ± 0.006
41	*Coriandrum sativum* L.	0.534 ± 0.047	1.785 ± 0.036	0.304 ± 0.098	0.002 ± 0.0004
42	*Foeniculum vulgare* Mill.	0.672 ± 0.036	1.221 ± 0.027	0.131 ± 0.012	0.005 ± 0.003
43	*Capparis spinosa* var. *canescens* Coss.	0.335 ± 0.024	1.035 ± 0.033	0.27 ± 0.042	0.02 ± 0.001
45	*Morus nigra* L.	0.757 ± 0.069	0.956 ± 0.036	0.093 ± 0.007	0.007 ± 0.005
47	*Capsella bursa-pastoris* Medik.	0.468 ± 0.044	1.125 ± 0.01	0.261 ± 0.055	0.014 ± 0.006
48	*Petroselinum crispum* (Mill.) Fuss	0.223 ± 0.038	0.968 ± 0.057	0.059 ± 1.185	0.011 ± 0.002
49	*Juglans regia* L.	0.813 ± 0.022	1.253 ± 0.036	0.204 ± 0.026	0.024 ± 0.004
51	*Salix babylonica* L.	0.971 ± 0.049	1.259 ± 0.035	0.091 ± 0.006	0.009 ± 0.001
52	*Malva sylvestris* L.	0.656 ± 0.038	1.279 ± 0.045	0.159 ± 0.017	0.01 ± 0.005
53	*Viscum album* subsp. *album* L.	0.597 ± 0.072	1.151 ± 0.039	0.219 ± 0.004	0.024 ± 0.002
54	*Mentha spica* subsp*. spicata* L.	1.825 ± 0.079	4.458 ± 1.238	0.062 ± 0.005	0.142 ± 0.009
55	*Cynara scolymus* L.	1.661 ± 0.129	1.654 ± 0.084	0.044 ± 0.003	0.028 ± 0.005

SC_50_ values for the 2,2-diphenyl-1-picrylhydrazyl radical scavenging method (DPPH), mM TE/mg for the cupric ion reducing antioxidant capacity assay (CUPRAC), mM FeSO_4_/mg for the ferric ion reducing antioxidant power assay (FRAP), and mg GAE/g for total phenolic content determination. GAE: gallic acid equivalent; TE: Trolox equivalent. Values were presented as mean ± standard deviation.

The antibacterial efficacy test conducted by AWDM revealed that only three samples examined exhibited antimicrobial activity against *Staphylococcus epidermidis* bacterial strains. The antimicrobial activity of the samples on other microorganisms has not been determined. The zone diameter values and MIC, MBC for the samples exhibiting antibacterial action are presented in Table 2.

**TABLE 2:** MBC, MIC and zone diameter values of samples against *Staphylococcus epidermidis*.

Sample no.	Sample name	Zone diameters (mm)	MIC (mg/mL)	MBC (mg/mL)
2	*Melissa officinalis L.*	10.07 ± 0.55	0.63	1.25
4	*Origanum vulgare subsp. hirtum (Link) Ietswaart*	7.90 ± 0.66	0.63	2.5
54	*Mentha spica* subsp*. spicata* L.	10.05 ± 0.18	0.63	1.25

* 5 mm groove diameter was deducted from zone diameters. MIC: Minimum inhibitory concentration, MBC: Minumum bactericidal concentration. SD: standard deviation.

The AWDM test for the antibacterial activity of the HTs revealed the largest zone diameters in *Melissa officinalis* (10.07 ± 0.55 mm) and *Mentha spica* subsp*. spicata* L. (10.05 ± 0.18 mm). The MIC values of samples exhibiting antimicrobial activity were found to be 0.63 mg/mL, while the MBC values ranged from 1.25 to 2.5 mg/mL (Table 2).

When the antioxidant and antimicrobial activity assays were evaluated collectively, the five samples exhibiting the highest biological activity were identified, in descending order, as *Melissa officinalis*, *Origanum vulgare* subsp. *hirtum* (Link) Ietswaart, *Origanum onites* L., *Mentha spica* subsp*. spicata* L., and *Lavandula* x *intermedia* Emeric ex Loisel.

The cytotoxic effects of different samples on A549, MCF7, and HEK293 cell lines were evaluated by IC_50_ values. The mean IC_50_ values and standard deviations of the cell lines used in the study are given in Table 3. It was determined that some HTs, such as *Origanum vulgare* subsp. *hirtum* (Link) Ietswaart (IC_50_: 99.68 ± 0.99 μg/mL for A549, 84.02 ± 2.05 μg/mL for MCF7, 125.93 ± 1.80 μg/mL for HEK293) and *Origanum onites* L. (IC_50_: A549: 132.89 ± 1.92 μg/mL, MCF7: 102.99 ± 3.56 μg/mL, HEK293: 208.23 ± 2.04 μg/mL), showed an effect close to colchicine, especially in the MCF7 cell line.

**TABLE 3:** IC_50_ values of the samples’ effects on the A549, MCF7, and HEK293 cell lines.

Sample no.	IC_50_ (µg/mL) (mean ± SD)		
	A549	MCF7	HEK293
2	157,16 ± 1,89	249,29 ± 2,80	419,90 ± 1,92
3	132,89 ± 1,92	102,99 ± 3,56	208,23 ± 2,04
4	99,68 ± 0,99	84,02 ± 2,05	125,93 ± 1,80
5	503,90 ± 2,48	408,28 ± 3,99	590,04 ± 1,88
7	390,44 ± 1,03	337,59 ± 2,57	370,66 ± 2,49
9	170,40 ± 1,45	293,66 ± 1,98	380,45 ± 1,90
11	312,99 ± 2,18	176,09 ± 2,05	208,22 ± 2,05
17	280,71 ± 1,39	304,11 ± 1,96	190,49 ± 1,45
18	299,16 ± 1,47	481,18 ± 3,02	349,02 ± 1,83
29	388,30 ± 2,76	474,30 ± 3,27	402,01 ± 2,05
37	440,29 ± 1,38	503,58 ± 2,85	483,47 ± 1,82
54	236,17 ± 1,36	130,42 ± 2,12	185,42 ± 1,39
Colchicine	10,49 ± 0,99	55,45 ± 1,11	23,33 ± 0,95

A549: Human lung cancer cell line, MCF7: human breast cancer cell line, HEK293: Human healthy embryonic kidney cell line, SD: standard deviation.

The mean and standard deviations of DNA_T_ (%) determined by the alkaline comet assay are given in Table 4. The greatest damage was observed in *Vitis viniferae L.* (60.15 ± 17.47) when the initial concentrations of all HTs were taken into account, while the least damage was observed in *Melissa officinalis* (28.54 ± 4.49). The third dosages of all HTs resulted in the highest damage in *Mentha spica* subsp*. spicata* L. (84.77 ± 12,26), and *Echinacea purpurea* (L.) *Moench* (86.88 ± 7.04), while *Melissa officinalis* (40.74 ± 9.79) sustained the least damage.

**TABLE 4:** DNA_T_ (%) obtained from the Comet Assay method compared with positive control.

Sample no.	First dose		Second dose		Third dose	
​	Sample concentration (µg/mL)	DNA_T_ (%) (Mean ± Sd)	Sample concentration (µg/mL)	DNA_T_ (%) (Mean ± Sd)	Sample concentration (µg/mL)	DNA_T_ (%) (Mean ± Sd)
2	8	28.54 ± 4.49	16	28.61 ± 4.11	32	40.74 ± 9.79
3	7	30.28 ± 5.09	14	38.99 ± 7.48	28	55.86 ± 18.44
4	5	56.26 ± 13.36	10	61.32 ± 11.42	20	76.16 ± 10.78*
5	25	39.93 ± 9.14	50	52.92 ± 13.22	100	75.01 ± 11.35*
7	20	44.51 ± 12.57^a^	40	44.93 ± 9.74^a^	80	63.42 ± 15.88^b^
9	9	44.15 ± 11.79	17	53.73 ± 13.33	34	60.07 ± 12.18
11	15	37.5 ± 4.77^a^	31	37.74 ± 5.2^a^	62	55.28 ± 9.35
17	14	35.32 ± 10.53	28	45.18 ± 13.55^a^	56	44.88 ± 10.72^a^
18	15	38.85 ± 9.4	30	43.07 ± 13.64	60	48.95 ± 18.11
29	20	60.15 ± 17.47	39	66.85 ± 14.64^b^	78	74.6 ± 16.53*
37	22	46.18 ± 8.38	44	70.28 ± 9.11*	88	86.88 ± 7.04*
54	12	48.06 ± 7.65	24	65.35 ± 16.58^b^	48	84.77 ± 12.26*
Control	21.15 ± 3.84					
Positive Control	66.43 ± 15.79					

Sd: Standart deviation. 400 cells were examined for each dose.

^a^ No difference was found between the doses administered to the botanical drug.

^b^ No significant difference was found with the positive control. *Significantly higher damage was detected compared to the positive control (p < 0,05).

Table 5 provides the mean and standard deviation of the number of micronucleus, NDI values, and percentages of the samples.- The analysis of micronucleus percentages in botanical drugs revealed that the greatest values were recorded in the third concentration of *Echinacea purpurea* (L.) *Moench* (11.05 ± 0.13). No binucleate cells containing micronucleus were found at the 1st concentration of *Origanum onites* L. The first dose of *Melissa officinalis* and the first and second doses of *Origanum onites* L. caused fewer micronuclei than the negative control, while the second and third doses of *Echinacea purpurea* (L.) *Moench* and the third dose of *Mentha spica* subsp*. spicata* L. caused more micronuclei than the positive control. In all samples except *Sambucus nigra* L. and *Hypericum perforatum* L., a dose-response relationship was determined between the number of micronucleus and the dose increase, compared to the negative and positive controls (p < 0.05).

**TABLE 5:** Micronucleus frequency and Nuclear Division Index of the tested samples.

​	Sample concentration (µg/mL)			Total micronucleus count (n = 4)			Micronucleus % ± SE			R	NDI ± SE		
Sample	First dose	Second dose	Third dose	First dose	Second dose	Third dose	First dose	Second dose	Third dose	​	First dose	Second dose	Third dose
2*	8	16	32	4	10	42	0.1 ± 0.04	0.25 ± 0.09	1.05 ± 0.21	0.90	1.55 ± 0.13	1.59 ± 0.19	1.59 ± 0.16
3	7	14	28	0	0	14	0 ± 0	0 ± 0	0.35 ± 0.1	0.84	1.6 ± 0.2	1.47 ± 0.13	1.54 ± 0.26
4*	5	10	20	40	68	135	1 ± 0.07	1.7 ± 0.17	3.38 ± 0.21	0.98	1.64 ± 0.15	1.54 ± 0.26	1.64 ± 0.21
5	25	50	100	12	82	128	0.3 ± 0.07	2.05 ± 0.23	3.2 ± 0.15	0.97	1.46 ± 0.08	1.57 ± 0.17	1.42 ± 0.15
7	20	40	80	36	41	40	0.9 ± 0.07	1.03 ± 0.1	1 ± 0.16	0.90	1.65 ± 0.06	1.5 ± 0.15	1.48 ± 0.16
9*	9	17	34	49	69	80	1.23 ± 0.23	1.73 ± 0.1	2 ± 0.11	0.98	1.46 ± 0.04	1.76 ± 0.1	1.53 ± 0.14
11*	15	31	62	7	29	53	0.18 ± 0.14	0.73 ± 0.28	1.33 ± 0.17	0.87	1.55 ± 0.17	1.59 ± 0.18	1.5 ± 0.06
17*	14	28	56	9	41	72	0.23 ± 0.1	1.03 ± 0.06	1.8 ± 0.23	0.97	1.54 ± 0.2	1.62 ± 0.14	1.56 ± 0.09
18*	15	30	60	5	12	33	0.13 ± 0.06	0.3 ± 0.11	0.83 ± 0.29	0.89	1.47 ± 0.07	1.5 ± 0.1	1.5 ± 0.13
29*	20	39	78	50	53	157	1.25 ± 0.18	1.33 ± 0.08	3.93 ± 0.14	0.93	1.68 ± 0.17	1.67 ± 0.12	1.48 ± 0.08
37*	22	44	88	135	257	442	3.38 ± 0.23	6.43 ± 0.17	11.05 ± 0.13	0.49	1.64 ± 0.07	1.51 ± 0.08	1.44 ± 0.13
54*	12	24	48	47	155	366	1,18 ± 0,14	3,88 ± 0,13	9,15 ± 0,19	0,63	1,58 ± 0,04	1,59 ± 0,12	1,48 ± 0,11
Positive Control	​			172			4.3 ± 0.20			—	1.32 ± 0.08		
Control	​	​	​	5			0.125 ± 0.01			—	1.72 ± 0.24		

* Dose-dependent change was observed according to linear regression p < 0.05. NDI: nuclear division index.

## 4 Discussion

Medicinal HTs have a history that extends over millennia and are prevalent throughout numerous cultures. Medicinal HTs are often perceived as intrinsically beneficial due to their historical usage and natural origins. Nonetheless, these plants must undergo toxicological and botanical evaluation to avert potential misuse (Shaw et al., 2012). The significance of this study is rooted in its 'from-shelf-to-cell’ methodology. Through the examination of products that have received pharmacopeial validation, we furnish essential data regarding the genotoxic safety, phytochemical characterization and biological activities of herbal teas, aspects sometimes neglected in solely botanical investigations of these recognized species.

A research in Turkiye indicated that the majority of botanical drugs marketed in herbal shops under traditional names and for particular purposes were inaccurately labeled (Kökçü et al., 2015). Similarly, some of the plants used in this study were mislabelled, and foreign matter (stones, glass, insects) was found in some packages. For instance, a botanical drug that was sold under the *Morus alba* label was mistakenly identified as *Morus nigra* during the diagnostic process. The antioxidant capacity and total phenolic metabolite content of these two species are distinct, as demonstrated by two distinct investigations (Polumackanycz et al., 2021; Abay and Eruygur, 2021). Due to the loss of structure in the teas sold, it has been difficult even for experts to diagnose some plants, and their identification has not been possible. Considering the general public, it is not surprising that people rely entirely on the information written on the label. This and similar situations are raising concerns that they could worsen the health of people seeking natural solutions for health problems. Therefore, it is deemed advantageous to implement stringent regulations and oversee the manufacturing, packaging, and marketing of therapeutic herbal teas to safeguard public health.

Analysis with the CUPRAC method demonstrated that *Melissa officinalis l., Althea officinalis l., Calendula officinalis l., Artemisia absinthium l., Petroselinum crispum (miller) a.w. hill.* exhibited higher capacity than those reported literature (Draginic et al., 2022; Apak et al., 2006; Xue et al., 2022; Ma et al., 2017; Saunoriūtė et al., 2023; Michalaki et al., 2023) while *Plantago lanceolata l.* showed lower capacity (Lukova et al., 2018). Additionally, both lower and higher efficacy have been reported for *Mentha spica l. Subsp. Spicata* have been reported in prior studies (Özer, 2018; Zengin et al., 2022). According to the results of the FRAP method, when the samples were compared with the literature, it was determined that *Origanum onis l., Origanum vulgare subsp. Hirtum (link) letswaart, Galega officinalis, Coriandrum sativum l., Morus nigra l., Petroselinum crispum (miller) a.w. hill Malva sylvestris l., and Cynara scolymus l.* showed higher capacity (Tang et al., 2013; Polumackanycz et al., 2021; Taskin and Sadikoglu, 2017; Sukhtezari et al., 2024; Eltablawy et al., 2015; Reza and Zeynab, 2011; Ben Salem et al., 2017) while *Sambucus nigra l., Rosa damascena mill., Plantago lanceolata l.,* and *Capparis ovata desf. Var. Canescens* showed lower capacity (Abay and Eruygur, 2021; Viapiana and Wesolowski, 2017; Trendafilova et al., 2023; Dalar et al., 2012). In addition, it was determined that *Salvia officinalis l. and Cichorium intybus l.* exhibited a similar level of capacity to the literature (Dalar et al., 2012; Farhat et al., 2014). In the DPPH assay, greater capacity was detected in *Lavandula intermedia emeric ex loisel. Althea officinalis l., Plantago lanceolata l., Salvia fruticosa miller, Urtica dioica l., Aronia melanocarpa (michx.) Elliott, Cichorium intybus l., Echinacea purpurea (L.) Moench, Galega officinalis, Foeniculum vulgare miller,* and *Juglans regia l.* (Xue et al., 2022; Blažeković et al., 2010; Beara et al., 2012; Dawra et al., 2023; Karneeb et al., 2023; Khare et al., 2012; Cvetanović et al., 2018; Abbas et al., 2014; Lepojević et al., 2017; Bednarska et al., 2020; Barros et al., 2009), while lower capacity was observed in *Origanum onis l., Origanum vulgare subsp., Hirtum (link) letswaart, Valeriana officinalis l, Vitis viniferae l., Rubus idaeus l.,* and *Capsella bursa-pastoris (L.) Medik.* than compared with other studies (Abay and Eruygur, 2021; Taskin and Sadikoglu, 2017; Ozkan et al., 2010; Elnaz et al., 2019; Fernandes et al., 2013; Veljkovic et al., 2018; Riaz et al., 2021; Grosso et al., 2011). However, while *Melissa officinalis l.* and *Salvia officinalis l*. showed similar levels of capacity (Farhat et al., 2014; Pereira et al., 2009; Koksal et al., 2011; Hamrouni-Sellami et al., 2013) both low and higher SC_50_ values were reported *Hypericum perforatum l., Rosa damascena mill., Salvia officinalis l., Calendula officinalis l., Artemisia absinthium l., Coriandrum sativum l.,* and *Malva sylvestris l.* comparison with previous studies (Tang et al., 2013; Reza and Zeynab, 2011; Trendafilova et al., 2023; Öztrk et al., 2009; Fathi et al., 2013; Özkan et al., 2004; Grzegorczyk et al., 2007; Kontogianni et al., 2013; Rigane et al., 2013; Preethi et al., 2006; Hbika et al., 2022; Bhat, 2018; Craciunescu et al., 2012; Msaada et al., 2017; Irfan et al., 2021; Mohammed et al., 2014; Shadid et al., 2021). Compared to similar studies in the literature for total phenolic content, higher levels were found in *Melissa officinalis l., Lavandula intermedia emeric ex loisel., Aronia melanocarpa (michx.) Elliott, Morus nigra l.,* and *Capsella bursa-pastoris (l.) Medik.* (Polumackanycz et al., 2021; Draginic et al., 2022; Cvetanović et al., 2018; Riaz et al., 2021; Dobros et al., 2022), while lower levels were detected in *Origanum vulgare subsp. Hirtum (link) letswaart, Hypericum perforatum l., Valeriana officinalis l., Rosa damascena mill., Lycium barbarum l., Vitis viniferae l., Crataegus aronia (l.) Bosc. Ex dc. Var. Aronia, Galega officinalis, Juglans regia l., Salix babylonica l., Malva sylvestris l.,* and *Cynara scolymus l.* (Taskin and Sadikoglu, 2017; Sukhtezari et al., 2024; Reza and Zeynab, 2011; Ben Salem et al., 2017; Viapiana and Wesolowski, 2017; Trendafilova et al., 2023; Elnaz et al., 2019; Fernandes et al., 2013; Öztrk et al., 2009; Fathi et al., 2013; Özkan et al., 2004; Irfan et al., 2021; Mohammed et al., 2014; Shadid et al., 2021; Baydar and Baydar, 2013; Mocan et al., 2014; Monagas et al., 2006; Khan et al., 2021; Do and Hwang, 2014; Hachkova et al., 2021; Almeida et al., 2008; Farag, 2023; Abdel Wahab et al., 2018). On the other hand, while similar levels were found in *Althea officinalis l., Rubus idaeus l.,* and *Mentha spica l. Subsp. Spicata* (Xue et al., 2022; Zengin et al., 2022; Veljkovic et al., 2018) *Origanum onis l., Sambucus nigra l., Tilia argentea desf. Ex dc., Plantago lanceolata l., Salvia fruticosa miller, Urtica dioica l., Calendula officinalis l., Artemisia absinthium l., Echinacea purpurea (l.) Moench, Coriandrum sativum l., Foeniculum vulgare miller, Capparis ovata desf. Var. Canescens,* and *Petroselinum crispum (miller) a.w. hill* reported values similar to some studies in the literature, lower than some studies, and higher than others (Michalaki et al., 2023; Taskin and Sadikoglu, 2017; Viapiana and Wesolowski, 2017; Dawra et al., 2023; Ozkan et al., 2010; Özkan and Özcan, 2018; Domínguez et al., 2020; Ayaz et al., 2020; Bahadori et al., 2020; Sanna et al., 2022; Laanet et al., 2024). A comparison of antioxidant capacity and total phenolic content with previously reported literature values is presented in Table 6. On the other hand, although these chemical assays provide essential data regarding the phytochemical profiling and reducing capacities of the samples in an *in vitro* setting, it is acknowledged that they do not directly reflect *in vivo* pharmacological outcomes or therapeutic efficacy.

**TABLE 6:** Comparison of antioxidant capacity and total phenolic content with values reported in the literature.

Sample no.	Sample	CUPRAC	FRAP	DPPH	TPC
2	*Melissa officinalis l.*	715,54 ± 4.79 μM TE/g (Draginic et al., 2022)↓	​	4,76 ± 0.48 μg/mL (Draginic et al., 2022) ↓, 32,9 ± 1.2 μg/mL (Pereira et al., 2009) ≈, 31,4 μg/mL (Koksal et al., 2011)≈	27,17 ± 0.51 mg GAE/g (Draginic et al., 2022)↓
3	*Origanum onis l.*	​	2,216 ± 0.007 (47)↓	0,32 ± 0.01 mg/mL (Taskin and Sadikoglu, 2017)↑, 807,5–395,75 μg/mL (Özkan et al., 2004)↑	54,57–141,80 mg GAE/g (161) ≈, 8,07 ± 0.94 mg GAE/mg (Taskin and Sadikoglu, 2017)↑, 106,13–149,40 mg GAE g (Özkan et al., 2004)↑
4	*Origanum vulgare subsp. Hirtum (link) letswaart*	​	2,199 ± 0.005 mm FeSO_4_/mg (Taskin and Sadikoglu, 2017)↓	0,22 ± 0.004 mg/mL (Taskin and Sadikoglu, 2017)↑	8,87 ± 1.0 mg GAE/mg (Taskin and Sadikoglu, 2017)↑
5	*Sambucus nigra l*	​	106,34 µmol Fe^2+^/100 g (Viapiana and Wesolowski, 2017)↑	​	252,4 mg GAE/g (Domínguez et al., 2020)↑, 15,23 mg GAE/g (Viapiana and Wesolowski, 2017)↓
7	*Hypericum perforatum l.*	​	​	4,71 ± 0.04 μg/mL (Öztrk et al., 2009) ↓, 96,0 ± 3.7 μg/mL (Fathi et al., 2013)↑	257,78 ± 1.82 mg GAE/g (Öztrk et al., 2009)↑ 505,7 ± 18 mg GAE/g (Fathi et al., 2013)↑
8	*Valeriana officinalis l.*	​	​	54,6–81,6 Μg/mL (Elnaz et al., 2019) ↑	312,3–390,6 mg GAE/g (Elnaz et al., 2019)↑
9	*Lavandula intermedia emeric ex loisel.*	​	​	17,17 ± 0.33 μg/mL (Blažeković et al., 2010)↓	15,55–30,79 mg GAE/g (Dobros et al., 2022)↓
11	*Rosa damascena mill.*	​	5,40 ± 0.14 mm Fe^2+^/g (Trendafilova et al., 2023)↑	0,27 ± 0.01 mg/mL (Trendafilova et al., 2023) ↑	248,97 ± 2.96 mg GAE/g (Özkan et al., 2004)↑, 212,19 ± 3.43 mg GAE/g (Trendafilova et al., 2023)↑, 211,92 ± 3.10 mg GAE/g (Baydar and Baydar, 2013)↑
12	*Tilia argentea desf. Ex dc.*	​	​	​	34,69–68,34 mg GAE/g (Ayaz et al., 2020)↑
13	*Althea officinalis l.*	0.05 Mmol TE/g (Apak et al., 2006)↓, 0,06 ± 0.00 Mm TE/µg (Decordier and Kirsch-Volders, 2006) ↓	​	140,3 ± 11,6 μg/mL (Xue et al., 2022)↓	1,8–175,8 mg GAE/g (Xue et al., 2022) ≈
16	*Plantago lanceolata l.*	21,9 ± 0.58 μm TE/g (Lukova et al., 2018)↑	201,4 ± 7.9 μmol Fe^2+^/g (44)↑	4,20 ± 0.19 μg/mL (Beara et al., 2012)↓	29 ± 0.2 mg GAE/g (Sanna et al., 2022)↑, 6,6 ± 0.3 mg GAE/g (Dalar et al., 2012)↓, 157,3 g GAE/kg (Sanna et al., 2022)↑, 23,4 ± 1.5 mg GAE/g (Laanet et al., 2024)↑
17	*Salvia fruticosa miller*	​	​	36,37 μg/mL (Dawra et al., 2023)↓, 12,9 ± 0.1 μg/mL (Karneeb et al., 2023)↓	42,98 ± 1.55 mg GAE/g (Dincer et al., 2012) ↓, 135,1 mg GAE/g (Dawra et al., 2023)↑, 12,57 g GAE/100 g (Karneeb et al., 2023) ↓
18	*Salvia officinalis l.*	​	112,87 ± 5,78–171,82 ± 8.10 mm Fe(ıı)/mg (Farhat et al., 2014)≈	13,49 ± 0,11–58,48 ± 1,17 (74) ≈,18,4 ± 5.5 μg/mL (Grzegorczyk et al., 2007)↓, 78,0 ± 1.7 μg/mL (Kontogianni et al., 2013)↑, 32,49 ± 2,26–55,91 ± 2.88 μg/mL (Farhat et al., 2014)≈	​
21	*Lycium barbarum l.*	​	​	​	61,59 ± 1.68 mg GAE/g (Mocan et al., 2014)↑
22	*Urtica dioica l.*	​	​	88,33 ± 2.88 μg/mL (Khare et al., 2012) ↓	​
23	*Aronia melanocarpa (michx.) Elliott*	​	​	32,40 ± 0.53 μg/mL (Cvetanović et al., 2018)↓	13,88 ± 0.02 mg GAE/g (Cvetanović et al., 2018)↓
24	*Cichorium intybus l.*	​	251,6 ± 9.7 μmol Fe^2+^/g (Dalar et al., 2012) ≈	67,2 ± 2.6 μg/mL (Abbas et al., 2014)↓	22,4 ± 0.3 mg GAE/g (Dalar et al., 2012)≈
26	*Calendula officinalis l.*	1,56 ± 0.02 Mmol TE/100 g (Ma et al., 2017)↓	​	0,35 ± 0,02 (80)↑, 100 μg/mL (Preethi et al., 2006)↓	109,27 ± 0.23 mg GAE/g (Rigane et al., 2013)↑, 116,92 ± 0.04 mg GAE/100 g (Ma et al., 2017)↓
29	*Vitis viniferae l.*	​	​	96–149 μg/mL (Fernandes et al., 2013)↑	112 ± 18 mg GAE/g (Monagas et al., 2006)↑, 95,32 ± 0.07 g GAE/μg (Khan et al., 2021)↑, 174 ± 17–550 ± 62 mg GAE/g (Fernandes et al., 2013)↑
32	*Artemisia absinthium l.*	105,04 ± 2,03–200,79 ± 0.57 μmol TE/g (42)↓	​	0.167 ± 0.004 mg/mL (Hbika et al., 2022)↑, 47,72 ± 1.21 μg/mL (Bhat, 2018)↓, 0,57 ± 0.05 mg/mL (Craciunescu et al., 2012)↑	123 ± 0.82 mg GAE/g (Preethi et al., 2006)↑, 69 mg GAE/g (Hbika et al., 2022)↓, 9,29 ± 0.51 mg GAE/g (Bhat, 2018)↓
33	*Crataegus aronia (L.) Bosc. Ex dc. Var. Aronia*	​	​	​	141,6 ± 0.9 mg GAE/g (Do and Hwang, 2014)↑
37	*Echinacea purpurea (L.) Moench*	​	​	0.527–0.919 g/mL (Lepojević et al., 2017)↓	1,69–3.24 g GAE/100 g (Lepojević et al., 2017)↑
39	*Rubus idaeus l.*	​	​	117,83 ± 1,49–199,18 ± 1.12 μg/mL (Veljkovic et al., 2018) ↑	59,68–96,83 mg GAE/g (Veljkovic et al., 2018)≈
40	*Galega officinalis*	​	558,18 ± 20,26 μmol FeSO_4_ g (Sukhtezari et al., 2024)↓	12,97 μg/mL (Bednarska et al., 2020) ↓	28,77 ± 2.47 mg (Hachkova et al., 2021) ↑, 81,05 ± 0.42 mg GAE/g (Sukhtezari et al., 2024)↑
41	*Coriandrum sativum l.*	​	0.136 ± 0.008 FeSO_4_ mmol/g (Tang et al., 2013)↓	32,00 ± 2.87 μg/mL (Msaada et al., 2017)↓, 1335,0 ± 37,7 dpph µg/mL (Tang et al., 2013)↑	0,94 ± 0.05 mg GAE/g (Msaada et al., 2017) ↓, 24,57 ± 0.70 mg GAE/g (Tang et al., 2013)↑
42	*Foeniculum vulgare miller*	​	​	12,16 ± 0.94 μg/mL (Barros et al., 2009)↓	8,61 ± 0.09 mg GAE/g (Barros et al., 2009)↑
43	*Capparis ovata desf. Var. Canescens*	​	​	586,8 ± 0.07 μg/mL (Abay and Eruygur, 2021)↑	79,9 ± 0.31 µg GAE/mg (Abay and Eruygur, 2021)↑
45	*Morus nigra l.*	​	6,42 ± 0.65 Fe^2+^/g (Polumackanycz et al., 2021) ↓	​	3,26 ± 0.75 mg GAE/g (Polumackanycz et al., 2021) ↓
47	*Capsella bursa-pastoris (L.) Medik.*	​	​	552,01 μg/mL (Riaz et al., 2021)↑, 420,96 μg/mL (Grosso et al., 2011)↑	1,56 × 10^−4^ mg GAE/g (Riaz et al., 2021), ↓
48	*Petroselinum crispum (miller) a.w. hill*	13,2 ± 0.6 nmol TE/g (Michalaki et al., 2023)↓	195,35 ± 5.03 mm Fe (ıı)/100 g (Eltablawy et al., 2015)↓	​	369,33 ± 15,5 mg GAE/g (Eltablawy et al., 2015)↑, 9,63 ± 2.60 mg GAE/g (Michalaki et al., 2023)↓
49	*Juglans regia l.*	​	​	​	270 ± 3 mg GAE/g (Almeida et al., 2008)↑
51	*Salix babylonica l.*	​	​	​	75,30 mg GAE/g (Farag, 2023)↑, 6,133–35,063 μg GAE/100 mg (Abdel Wahab et al., 2018)↑
52	*Malva sylvestris l.*	​	0.149 ± 0.001 mmol/g (Reza and Zeynab, 2011)↓	22,11 μg/mL (Irfan et al., 2021)↑, 33,35 ± 0.76 μg/mL (Shadid et al., 2021)↓, 16,691 μg/mL (Mohammed et al., 2014)↓, 0.077 ± 0.002 mg/mL (Ma et al., 2017)↑	40,91 ± 19 mg GAE/g (Irfan et al., 2021) ↑, 23,35 ± 0.76 mg GAE/g (Shadid et al., 2021)↑, 24,123 ± 0.718 mg GAE/g (Mohammed et al., 2014)↑, 15,11 ± 0.28 mg GAE/g (Reza and Zeynab, 2011)↑
53	*Viscum album l. Subsp. Album*	​	​	​	33,789 ± 1,606 mg GAE/g (Pietrzak and Nowak, 2021) ≈
54	*Mentha spica l. Subsp. Spicata*	2,36 ± 0.09 mM TE/g (Özer, 2018) ↓, 37,72 ± 0.81 mM TE/mg (Zengin et al., 2022)↑	​	​	27,39 ± 0,95–142,62 ± 3.52 mg GAE/g ≈ (Zengin et al., 2022)
55	*Cynara scolymus l.*	​	315,91 ± 8.36 μmol Fe (ıı)/g (Ben Salem et al., 2017)↓	​	49,49 ± 0.39 mg GAE/g (Ben Salem et al., 2017) ↑

When comparing the results with the literature, the symbols ≈, ↑, and ↓ were used to indicate *similar to the literature*, *higher than the literature*, and *lower than the literature*, respectively. In the conducted study, the results were expressed as follows: SC_50_ values for the 2,2-diphenyl-1-picrylhydrazyl radical scavenging method (DPPH), mM TE/mg for the cupric ion reducing antioxidant capacity assay (CUPRAC), mM FeSO_4_/mg for the ferric ion reducing antioxidant power assay (FRAP), and mg GAE/g for total phenolic content determination. GAE: gallic acid equivalent; TE: Trolox equivalent. Values were presented as mean ± standard deviation.

*Melissa officinalis*, consistent with the diagnosis, and was determined to be the HT with the highest capacity when all antioxidant capacity determination methods were evaluated together. It is known to have a high antioxidant effect, primarily due to the phenolic metabolites it contains, such as citronellal and neral (M et al., 2017).

Nasungan (2022) demonstrated that ethanolic extracts of some wild tea leaves significantly inhibited the growth of these bacterial strains using the agar-well diffusion method, supporting the antibacterial potential of phenolic- and flavonoid-rich plant extracts (Antioxidant, 2022). While the vast majority of samples in this study did not show antimicrobial activity, only *Melissa officinalis L*., *Origanum vulgare subsp. Hirtum (Link) Letswaart* and, *Mentha spica* subsp*. spicata* L were effective against a single bacterial strain (*Staphylococcus epidermidis*) (Table 2). These findings can be ascribed to the relatively modest concentration (5 mg/mL) and the use of water instead of strong organic solvents present in the tea-based extracts prepared for daily use.

It was determined that the aqueous solution prepared after methanol extraction of *Melissa officinalis*, did not show activity against, similar to this study, *E. coli* and *P. aeruginosa* but did show activity against *S. aureus*, with an MIC value of 1.25 mg/mL (Čanadanović-Brunet et al., 2008). Another investigation using ethanol extracts of *Melissa officinalis*, demonstrated effectiveness against *P. aeruginosa* and *C. albicans*, with MIC values of 28.80 μg/mL and 25.60 μg/mL, respectively, and ineffectiveness against *S. aureus, E. coli*, and *K. pneumoniae*, as in this study (Abdel-Naime et al., 2019). It has been determined that the, similar to this study, extract obtained by water infusion of the *Melissa officinalis*, exhibits antimicrobial activity against *K. pneumoniae, S. aureus, P. aeruginosa*, and *E. coli* bacterial strains (MIC: 5 mg/mL, 10 mg/mL, 20 mg/mL, and 5 mg/mL) (Stefanović and Comic, 2012). In a study comparing the antimicrobial activity of methanol extract *of Melissa officinalis* grown under natural and *in vitro* conditions, it was observed that the extract from plants grown only under *in vitro* conditions showed activity against bacterial strains of *S. epidermidis, S. aureus, S. pyogenes, E. coli, Salmonella typhimurium, P. aeruginosa, P. vulgaris, E. coli, Klebsiella pneumoniae*, and *S. pyogenes* (Ulgen et al., 2023). Albayrak et al. (Albayrak et al., 2013) in 2013 conducted a study, which used *Melissa officinalis* extracts prepared with 4 different solvents, the antimicrobial activity against *K. pneumoniae, P. aeruginosa,* and *S. aureus* strains was examined using the AWDM. The results of that study showed that water infusion did not exhibit any activity, while the methanol extract produced a 8 mm zone against *P. aeruginosa*.

The same investigation by Albayrak et al., extracts of *Thymus vulgaris* were also examined, and it was found that water infusion had no antimicrobial activity, while the methanol extract formed a 10 mm zone against *P. aeruginosa*. Another study using a ready-made extract of *Thymus vulgaris* found no antimicrobial effect against *S. aureus*, *S. mutans*, or *P. aeruginosa*, but the extract inhibited *C. albicans* with an MIC value of 50 mg/mL (de Oliveira et al., 2017). Ethanolic *Origanum onites* extract was active against *P. aeruginosa, E. coli*, and *S. aureus*, each with an MIC of 25 μg/mL (Coccimiglio et al., 2016). Comparative research on *Origanum onites* obtained by maceration versus supercritical fluid extraction showed both methods effective against *S. aureus*, *E. coli*, and *C. albicans*, though supercritical fluid extraction yielded the strongest activity (Žitek et al., 2021).

*Mentha spicata*, crude extract proved inactive against *S. aureus* and *E. coli*, while its essential oil exhibited inhibitory properties (Scherer et al., 2013). Qadir et al. (2017) assessed *Mentha spicata* by disk diffusion and reported no effect on *E. coli* but minimal activity against *S. aureus* (Abdul Qadir et al., 2017). The methanol extracts of *Mentha spicata* collected from various locations in Pakistan exhibited antimicrobial activity with MIC values ranging from 0.25 mg/mL to 0.50 mg/mL against *S. aureus,* 0.125 mg/mL to 2.0 mg/mL against *E. coli*, and 0.06 mg/mL to 0.5 mg/mL against *P. Aeruginosa* (Ullah, 2012). In a study using ethanol extract and different fractions, activity was observed against *S. aureus* and *E. coli* bacteria in the disk diffusion method, while no activity was seen against *P. vulgaris* and *K. pneumoniae* bacteria (Arumugam et al., 2010).

Tea infusions of *Melissa officinalis*, *Mentha spicata*, and *Salvia officinalis*, inhibited *S. aureus* with MICs of 0.5, 0.5, and 0.25 mg/mL, and MBCs of 1, 1, and 0.5 mg/mL, respectively (Silva et al., 2023).

Combined assessments of *Melissa officinalis* and *Thymus vulgaris* reported that these two plants caused a decrease in Nuclear Factor-κB reactivity, while leading to an increase in the number of apoptotic cells and higher Tumor Necrosis Factor alpha and Interleukin-6 immunoreactivity, thus causing damage to cancer cells (Jahanban-Esfahlan et al., 2015). *Melissa officinalis* ethanol extract IC_50_ value was 301.4 ± 10.26 μg/mL in another study (Moacǎ et al., 2018).

A study utilizing the methanol extract of *Origanum vulgare* reported IC_50_ values ranging from 140.77 ± 2.13 to 109.51 ± 1.28 μg/mL following 24–72 h of exposure to the HCT-116 cell line (Grbović et al., 2013). A separate study involving an extract of *Origanum vulgare* reported an IC_50_ value of >500 μg/mL for the A549 cell line and 10.76 ± 0.8 μg/mL for the MCF7 cell line (Erenler et al., 2023).

In a study using hypericin, the active metabolite of *Hypericum perforatum L*., no cytotoxic effect was observed on healthy cell lines, while cytotoxic activity was observed on cancer cell lines (Mirmalek et al., 2016). In a study conducted on the A549 cell line using the methanol extract of *Hypericum perforatum L*., the IC_50_ value was found to be 93.22 ± 12.28 μg/mL (Matić et al., 2021).

A study investigating the impact of *Rosa damascena* ethanol extract on the HeLA cancer cell line yielded IC_50_ values of 2,135 μg/mL, 1,540 μg/mL, and 305.1 μg/mL after 24, 48, and 72 h of exposure, respectively (Zamiri-Akhlaghi et al., 2011). A separate investigation utilizing the ready-to-use powder extract of *Rosa damascena* revealed an IC_50_ value of 375 μg/mL in healthy fibroblast cells (Trendafilova et al., 2023). The value was determined to be lower across all cell lines in this study.

In a study conducted to investigate the effect of *Salvia fruticosa* extracts prepared with different solvents on different cell lines, it was determined that IC_50_ values varied for both parameters, and the IC_50_ values of the ethanol extract were significantly lower than in this study (14.6 ± 6.1 μg/mL in the HCT-116 cell line, 31.1 ± 0.3 μg/mL in the Caco-2 cell line) (Dawra et al., 2023). In another study using a methanol extract of *Salvia fruticosa*, it was found that the IC_50_ values were also quite low compared to this study, with an IC_50_ value of 57.95 μg/mL for the melanoma cancer line and 72.67 μg/mL for the healthy skin fibroblast line (Koutsoulas et al., 2019).

While no cytotoxic effect was observed on cancer cells after 24-h exposure to different concentrations (0–2000 μg/mL) of *Echinacea purpurea* ethanol extract, 48-h exposure was found to cause cytotoxicity (Tsai et al., 2012). In a study using the HeLa cervical cancer cell line, it was found that extracts prepared with different solvents had IC_50_ values ranging from 73 to 928 μg/mL after 24 h of exposure, with the highest IC_50_ value belonging to the methanol extract and the water extract having an IC_50_ value similar to this studie’s (578 μg/mL) (Mohamed Sharif et al., 2021).

In a study similar to this study, using extracts of *Mentha spicata, Salvia officinalis*, and *Melissa officinalis* obtained by lyophilization after water infusion, it was found that the IC_50_ values of the extracts against the MCF7 cell line were 203 ± 1.50 μg/mL, 198 ± 0.97 μg/mL, and 239 ± 0.99 μg/mL, respectively, which is consistent with this studie’s results (Silva et al., 2023).

Polyphenols found in medicinal plants can inhibit the proliferation of cancer cells through various pathways, including the suppression of Nuclear factor-kB (NF-kB), activator protein-1 (AP-1) transcription factor, protein kinases, and growth factor receptor (GFR)-mediated pathways, cell cycle arrest, and the induction of apoptosis. These events are related to the amount and variety of polyphenols they contain (Ghasemi et al., 2021). In most of this study, the IC_50_ value for cancer cells was found to be lower than the IC_50_ value for healthy cell lines, meaning that the plants were more effective against cancer cell lines than against healthy cell lines. However, this was different for some plants, or no significant difference was observed. This could be because the MTT test works through mitochondrial activity, and normal cell lines have lower mitochondrial activity than cancer cells (Erenler et al., 2023; Ghasemi et al., 2021).

In a study using an ethanol extract of *Melissa officinalis*, concentrations between 10 and 150 μg/mL were examined using the Comet assay and found to show no genotoxic activity on leukocytes (Kamdem et al., 2013). Tests conducted on animals with oral administration at different doses also showed no genotoxic activity (de Carvalho et al., 2011; Lobach et al., 2024). Although this study showed an increase in DNA damage compared to the control in both the Comet assay and the micronucleus assay, the lowest average DNA_T_ (%) was observed in *Melissa officinalis*, and the increase in micronucleus count was negligible.

In a study where the methanol extract of *Salvia fruticosa* was tested on human lymphocyte cells, it was determined that the application of 1.5 μg/mL, 3 μg/mL, and 6 μg/mL concentrations for 24 h resulted in an average of 0.8%, 0.6%, and 1% micronucleus formation, respectively, and that treatment for 48 h resulted in an average of 0.72%, 0.92%, and 0.90% micronucleus formation, respectively (Sevindik and Rencuzogullari, 2014). Similar percentages of micronucleus formation were observed in this study as well.

Concentrations of 250, 500, and 1,000 μg/mL of the distilled water extract of *Salvia officinalis* prepared using a Soxhlet apparatus resulted in a decrease in micronucleus count compared to the control (Ad’hiah et al., 2011). The extract of *Salvia officinalis*, prepared by lyophilization after a 90-min maceration, did not cause a change in micronucleus count at concentrations of 0.075–1.25 mg/mL (Šobot et al., 2024). In this study, it was determined that, in line with the literature, it did not create a significant difference compared to the control group.

In this study, when the results of the Micronucleus Assay and the Comet Assay are compared within themselves, there is a noticeable similarity between them. In both methods, an increase in damage was observed with an increase in dose. Nevertheless, the results obtained from the Comet Assay were found to detect higher damage compared to the Micronucleus Assay. The Comet Assay directly visualizes DNA damage individually, measuring the damage quantitatively. The Micronucleus Assay, on the other hand, measures changes in non-dividing chromosome fragments during cytokinesis, which result from larger changes at the chromosomal level. The difference between this results may have stemmed from this.

The results of similar studies in the literature differ both from each other and from the data obtained in this study. These differences can stem from laboratory conditions such as the extraction methods used (Elnaz et al., 2019; Domínguez et al., 2020; Čanadanović-Brunet et al., 2008; Abdel-Naime et al., 2019; Adham et al., 2021; Vlaisavljević et al., 2019; Eroǧlu et al., 2010), and the solvent temperatures (Baydar and Baydar, 2013), as well as environmental factors such as whether the plant is fresh or dried (Özkan et al., 2004), whether it is harvested in different months (Dalar et al., 2012; Farhat et al., 2014; Ozkan et al., 2010), and whether it is grown in cultivation or in the wild (Dincer et al., 2012). Additionally, the use of different parts of the plant (Kontogianni et al., 2013), drying methods (Hamrouni-Sellami et al., 2013), soil composition (Khan et al., 2021), and environmental variables such as light intensity, temperature, relative humidity, and wind speed (ULGEN et al., 2023; Neagu et al., 2019) can lead to differences in antioxidant capacity and total phenolic content. Specifically, the growth of parasitic plants like mistletoe on different hosts (Pietrzak and Nowak, 2021) can cause significant changes in their composition. Additionally, the fact that the concentrations used in this study even lower than the MIC values reported in the literature may have affected results (Stefanović and Comic, 2012; Albayrak et al., 2013). The application of different exposure durations (de Oliveira et al., 2017; Abdul Qadir et al., 2017), and the use of different cell lines in the studies (Moacǎ et al., 2018) may have also led to this difference, as some of the studies in the literature were conducted with oral administration (İla and Timoçin, 2020; Peron et al., 2013). Additionally, the fact that the results of some studies are reported based on dried plant weight, while others are based on extract weight, makes direct comparison difficult. The use of different determination methods and the expression of results in different units can also contribute to this diversity in the literature. Türkiye’s geographical characteristics may also have caused differences between the literature and the results through these factors.

While the safety of a drug must be proven before it is licensed, for herbal medicinal products, it is sufficient for licensing if it can be bibliographically proven that the product has been used for at least 15 years in Türkiye and EU member states, and for 30 years in other countries. So, these products are considered safe unless proven otherwise in terms of safety. In fact, the monograph published in 2018 by the European Committee on Herbal Medicinal Products includes plants used for many different indications, including those in this study, and pre-clinical and clinical safety studies are not considered necessary for these herbal medicinal products, which are used medically and considered safe under Directive 2001/83/EC (Türkiye İlaç ve Tıbbi Cihaz Kurumu (TİTCK), 2023; European Union Publications Office, 2013). Therefore, genotoxicity studies using herbal teas prepared similarly to our study are quite limited in the literature.

## 5 Conclusion

Inconclusion some HTs were found to exhibit high antioxidant capacity, substantial phenolic content, antimicrobial activity, high cytotoxicity on cancer cell lines, low cytotoxicity on healthy cell lines, and low genotoxicity. Conversely, HTs with opposing properties were also identified.

The study identified *Melissa officinalis L., Origanum onites L.*, *Origanum vulgare subsp. Hirtum (Link) Letswaart*, *Lavandula intermedia Emeric Ex Loisel.*, and *Mentha spica L. Subsp. spicata* as the five plants with the highest antioxidant capacity, while *Melissa officinalis L., Origanum vulgare subsp. Hirtum (Link) Letswaart*, and *Mentha spica L. Subsp. spicata* exhibited antimicrobial activity against *Staphylococcus epidermidis*. *Melissa officinalis L.,* and *Lavandula intermedia Emeric Ex Loisel.* demonstrated high cytotoxicity in cancer cell lines but relatively lower cytotoxicity in healthy cell lines. In contrast, *Origanum onites L.* and *Origanum vulgare subsp. Hirtum (Link) Letswaart* showed high cytotoxic activity across all cell lines, whereas *Mentha spica L. Subsp. spicata* exhibited increased cytotoxicity in healthy cell lines. Furthermore, *Origanum vulgare subsp. Hirtum (Link) Letswaart* and *Mentha spica L. Subsp. spicata* induced higher DNA damage than positive control at their final doses in the comet assay. A dose-dependent increase in micronucleus formation was observed in samples *Origanum vulgare subsp. Hirtum (Link) Letswaart*, *Lavandula intermedia Emeric Ex Loisel.*, and *Mentha spica L. Subsp. spicata*.

Therefore, the findings indicate that *Melissa officinalis L.,* is the most promising therapeutic candidate due to its high antioxidant capacity, selective cytotoxicity toward cancer cells, and antimicrobial activity. Conversely, the pronounced cytotoxicity of *Origanum onites L.* and *Origanum vulgare subsp. Hirtum (Link) Letswaart* across all cell lines, along with the elevated cytotoxicity, DNA damage, and significant increases in genotoxic markers (as evidenced by comet and micronucleus assays) in the healthy cell line treated with *Mentha spica L. Subsp. spicata*, suggest potential genotoxic risks associated with these samples. The dose-dependent increase in micronuclei observed in samples *Origanum vulgare subsp. Hirtum (Link) Letswaart*, *Lavandula intermedia Emeric Ex Loisel.*, and *Mentha spica L. Subsp. spicata* further supports this concern. Consequently, the biological effects of these samples should be further elucidated through mechanism-based advanced studies, and their safety profiles, particularly with respect to genotoxicity, should be validated using *in vivo* models.

Herbal teas have been used by people for many years to maintain health, prevent diseases, and treat illnesses. Herbal teas appear advantageous due to their easy accessibility, affordability compared to medications, and lower potential for side effects. However, the literature examining the positive and negative effects of herbal teas grown in Türkiye on health is limited. It is believed that focusing particularly on the positive aspects of herbal teas could lead to overlooking potential negative effects. In addition, the consumption of medicinal plants that are believed to have a positive effect on health but for which there are not enough studies in the literature, or which cannot demonstrate their effectiveness in the literature when grown under different conditions, will not only delay the healing of diseases but can also harm the individual and society economically.

The inability to conduct *in vivo* experiments, failure to obtain suitable samples for the herbarium during the botanical identification of herbal teas constitute the limitations of our study. On the other hand, the fact that no other study in the literature has examined such a diverse range of herbal teas obtained from a single source simultaneously using these experimental methods constitutes its strength.

## Data Availability

The raw data supporting the conclusions of this article will be made available by the authors, without undue reservation.
