# Supplementary material for: From cup to cell: phytochemical characterization and safety profiles of some herbal teas in Türkiye
Source: Front Pharmacol. 2026 Apr 28;17:1803892. doi: 10.3389/fphar.2026.1803892 (PMC13161097; doi:10.3389/fphar.2026.1803892)
Supplement: Supplementary file 1 [file Supplementaryfile1.docx]

Supplementary Material

# Supplementary Data

**Utilized Chemicals and Their Preparation**

**Copper (II) ion reducing antioxidant capacity (CUPRAC)**

CuCl_2_ (10 mM) (sigma): 1.7048 g CuCl_2_‚2H_2_O, dissolved in 1 L distilled water.

Neocuproine (7,5 mM) (sigma): 1,56 g Neocuproine, dissolved in 1 L %96 ethanol.

Ammonium acetate (1 M) (sigma): 77,08 g C_2_H_7_NO_2_ dissolved in 1 L distilled water.

Trolox (2 mM): 0,05 g Trolox, dissolved in 1 L %75 ethanol.

Ethanol (%75) (Merck)

**2,2-diphenyl-1-picrylhydrazyl radical scavenging activity determination (DPPH)**

Ascorbic acid (sigma): 1 mg/mL stock solution was prepared, and then working solutions in the range of 0.05-0.8 mg/mL were prepared.

Butylated Hydroxytoluene (BTT) (sigma): 1 mg/mL stock solution was prepared, and then working solutions in the range of 0.05-0.8 mg/mL were prepared.

2,2-diphenyl-1-picrylhydrazyl (DPPH) (0,1 mM): 3.94 mg was dissolved in 100 mL of methanol.

**Ferric reducing antioxidant power determination (FRAP)**

Ascorbic acid (sigma): 1 mg/mL stock solution was prepared, and then working solutions in the range of 0.05-0.8 mg/mL were prepared.

Acetate Buffer (pH 3.6) (300 mM): 1.55 g of Sodium Acetate Trihydrate (Sigma (C_2_H_3_NaO_2_⋅3H_2_O)) was dissolved in 8 mL of acetic acid (Sigma (C_2_H_4_O_2_)) and the final volume was adjusted to 100 mL with distilled water.

HCl (40 mM): 340 µL Hydrochloric acid (HCl) was prepared by adding 340 µL to 100 mL of distilled water.

2,3,5-Triphenyltetrazolium Chloride solution (10 mM): Prepared by dissolving 0.0309 g of 2,4,6-Tris(2-pyridyl)-s-triazine (TPTZ) (Merck) in 40 mM HCl solution to a final concentration of 10 mL.

FeCI_3_⋅6H_2_O (sigma) (20 mM): 0.0540 g of Iron (III) Chloride Hexahydrate (FeCI_3_⋅6H_2_O) was dissolved in distilled water to a final concentration of 10 mL.

FeSO_4_⋅7H_2_O (1 mM): 278.05 mg of Ferrous Sulfate Heptahydrate was diluted with 100 mL of distilled water to prepare the solution.

FRAP Reagent: It was freshly prepared by mixing 2.5 mL of 2,3,5-Triphenyltetrazolium Chloride solution, 25 mL of acetate buffer, and 2.5 mL of FeCI_3_⋅6H_2_O solution, and incubating the mixture at 37 ^o^C for 30 minutes.

**Total phenolic content determination**

Folin-Ciocalteu reagent: Obtained ready-made. It was diluted 1/3 with distilled water.

Sodium Carbonate (2%): 2 g Sodium Carbonate (NaCO_3_) was dissolved in distilled water to a final concentration of 100 mL.

Gallic acid: 1 mg/mL stock solution was prepared, and then working solutions in the range of 0.05-0.8 mg/mL were prepared.

Ethanol (70%): 70 units of ethanol were mixed 30 units of distilled water.

Physiological saline (0.85%): 8.5 grams of NaCl (sodium chloride) were dissolved in 1 L of distilled water.

**Antimicrobial activity**

McFarland Standard Solution - 0.5 MFU: Purchased ready-made.

McFarland Standard Solution - 1.0 MFU: Purchased ready-made.

Physiological saline

Mueller-Hinton Agar (MHA): 34 g of powdered medium was dissolved in 1 L of distilled water and autoclaved at 121°C for 15 minutes to prepare.

5% Sheep Blood Mueller-Hinton Agar: The prepared MHA was cooled to 45-50°C and prepared by adding 5% defibrinated sheep blood by volume.

Cation-adjusted Mueller Hinton broth (CAMHB): 34 g of powdered broth was dissolved in 1 L of distilled water and autoclaved at 121°C for 15 minutes to prepare.

Tryptic Soy Agar: 40 g of powdered nutrient medium was dissolved in 1 L of distilled water and autoclaved at 121°C for 15 minutes to prepare.

Sabouraud Dextrose Agar: 65 g of powdered medium was dissolved in 1 L of distilled water and autoclaved at 121°C for 15 minutes to prepare.

Amphotericin B (İbrahim Ethem Ulagay İlaç Sanayii A.Ş.)

Meropenem (İbrahim Ethem Ulagay İlaç Sanayii A.Ş.)

**Cell line studies**

DMEM F-12 (Gibco)

DMEM with high glucose (Gibco)

Fetal Bovine Serume (FBS) (Gibco)

Penicillin/Streptomycin (Gibco)

L-Glutamine (Gibco)

DMSO (Merck)

Ethanol (Merck)

Trypan blue (Sigma)

Phosphate buffered saline (PBS) (Gibco)

Trypsin (Gibco)

MTT solution (Sigma)

Ethylenediaminetetraacetic acid (EDTA) (Gibco)

Acetic acid

Isopropyl alcohol (Merck)

Preparation of the mediums

Mediums were purchased commercially ready-to-use (500 mL). It was prepared by adding 10% (50 mL) FBS, 1% antibiotic/antimycotic (5 mL), and 1% L-Glutamine (5 mL).

DMEM F-12

DMEM high glucose

%10 FBS : 50 mL

%1 Penicillin/streptomycin: 5 mL

%1 L-Glutamine : 5 mL

Preparation of MTT solution

The MTT stock solution was prepared by dissolving it in PBS at a concentration of 5 mg/mL.

**Alkali comet assay**

Hydrochloric acid HCl (5 N): 91.25 mL of concentrated HCl was diluted with distilled water to a final volume of 500 mL.

Sodium Hydroxide (NaOH) (AFG SCIENTIFIC) (10 M): 400 g of NaOH pellets were dissolved in distilled water to a final volume of 1 L.

Ethanol (Merck)

Low melting agarose (LMA) (%0,65) (Sigma Aldrich): 0,65 g LMA dissolved in 100 mL PBS.

High melting agarose (HMA) (Sigma Aldrich): 0,65 g HMA dissolved in 100 mL distilleted water.

Histopaque-1077 (Sigma Aldrich)

PBS (Thermo Fischer Scientific)

Lysis Solution Stock: 146.10 g NaCl, 37.2 g EDTA, and 1.2 g Tris were dissolved in distilled water, adjusting the pH to a final value of 10 using NaOH and HCl. The final volume of the solution was brought up to 1 L with distilled water.

DMSO (Merck)

Triton X (AFG SCIENTIFIC)

Tris Base (AFG SCIENTIFIC)

EDTA (0.2 M) (Bio Basic): 37.2 g of EDTA was dissolved in distilled water to a final volume of 500 mL.

Neutralization buffer (10 M): Prepared by dissolving 48.5 g of Tris in distilled water, adjusting with HCl to a final pH of 7.5, and bringing the volume to 1 L.

Electrophoresis buffer: Freshly prepared by mixing 37.5 mL of NaOH solution and 6.75 mL of EDTA solution, and bringing the volume to 1250 mL with distilled water.

Lysis solution: For each Hellendahl staining dish, 89 mL of stock lysis solution, 10 mL of DMSO, and 1 mL of Triton-X were mixed and prepared fresh, placed in foil-covered staining dish to protect from light, and refrigerated.

Ethidium Bromide (AFG BIOSCIENCE): 5 mg Ethidium Bromide was prepared by dissolving in 50 mL of distilled water.

Hydrogen Peroxide (H_2_O_2_) Stock: 11.5 µL of 30% hydrogen peroxide was prepared by adding distilled water to a final volume of 1 mL.

**Micro nucleus assay**

LymphoPrime 2 (Capricorn Scientific): Purchased ready-made.

DMSO (Merck)

Buffer A (KH_2_PO_4_): 11.34 g of KH_2_PO_4_ was dissolved in 250 mL of distilled water to prepare the buffer.

Buffer B (Na_2_HPO_4_∙2H_2_O): 7.37 g was dissolved in 250 mL of distilled water and prepared.

Giemsa Azure Methylene Blue Stain (Merck)

Nitric Acid (HNO3) (70%): 68.75 mL of concentrated Nitric acid was added to distilled water to bring the volume to 1 L.

Mitomycin C (200 µg/mL) (Cayman Chemicals): 5 mg was dissolved in 25 mL of ultra-distillited water. During the study, it was used diluted 1/10 with ultra- distillited water.

Cytochalasin B (600 µg/mL) (Cayman Chemicals): 5 mg of Cytochalasin B was dissolved in 8.3 mL of DMSO to prepare the solution. During the study, it was used diluted 1/10 with sterile medium.

Potassium chloride (KCl) (0.075 M) (Merck): 1.397 g of KCl was dissolved in 250 mL of distilled water.

Formaldehyde (Merck)

Acetic Acid (Glacial) (Isolab)

Methanol (Merck)

Ethanol (70%) (Merck)

Fixation solution: Prepared by adding 1 part acetic acid to 3 parts methanol.

Stain: 10 mL of Buffer A, 10 mL of Buffer B, and 10 mL of Giemsa stain were mixed with 170 mL of distilled water to prepare the stain.

# Supplementary Figures and Tables

Supplementary Table 1

| **Sample No.** | **Voucher No** | **Turkish name on the label** | **Common names in English corresponding to the Turkish name on the package** | **The botanical name on the label** | **The identified botanical names of the samples** |
| --- | --- | --- | --- | --- | --- |
| 1* | EB-1 | Tıbbi nane | Peppermint | *Mentha piperita* | *Nepeta* sp. (not suitable for identification at the species level) |
| 2 | EB-2 | Melisa | Lemon balm | *Melissa officinalis* | *Melissa officinalis* L. |
| 3 | EB-3 | Bilyalı kekik | Cretan oregano | *Origanum onites* | *Origanum onites* L. |
| 4 | EB-4 | Tıbbi kekik (İstanbul kekik) | German thyme | *Thymus vulgaris* | *Origanum vulgare* subsp. *hirtum* (Link) Ietswaart |
| 5 | EB-5 | Kara mürver çiçeği | European elderberry flower | *Sambucus nigra* | *Sambucus nigra* L. |
| 6* | EB-6 | Kara mürver meyvesi | European elderberry fruit | *Sambucus nigra fructus* | *Sambucus* sp. (not suitable for identification at the species level) |
| 7 | EB-7 | Sarı kantaron | St John's wort | *Hypericum perforatum* | *Hypericum perforatum* L. |
| 8 | EB-8 | Kedi otu | Valerian | *Valeriana officinalis* | *Valeriana officinalis* L*.* |
| 9 | EB-9 | Lavanta | Lavender | *Lavandula intermedia* | *Lavandula* x *intermedia* Emeric ex Loisel. |
| 10 | EB-10 | Tıbbi papatya | Chamomile | *Matricaria recutita* L | *Matricaria chamomilla* var. *recutita* (L.) Fiori |
| 11 | EB-11 | Gül çayı | Damask rose | *Rosa damascena* | *Rosa damascena* Mill. |
| 12 | EB-12 | Ihlamur | Linden | *Tiliae flos* | *Tilia tomentosa* Moench |
| 13 | EB-13 | Tıbbi hatmi | Marshmallow | *Althaea officinalis* | *Althaea officinalis* L. |
| 14* | EB-14 | Gülhatmi | Common hollyhock | *Althea rosea* | *Althaea* (not suitable for identification at the species level) |
| 15* | EB-15 | Sığırkuyruğu | Mullein | *Verbascum asperuloides* | *Verbascum* sp. (not suitable for identification at the species level) |
| 16 | EB-16 | Sinirli ot | Ribwort plantain | *Plantago lanceolata* | *Plantago lanceolata* L. |
| 17 | EB-17 | Anadolu ada çayı | Greek sage | *Salvia triloba* | *Salvia fruticosa* Mill. |
| 18 | EB-18 | Tıbbi adaçayı | Sage | *Salvia officinalis* | *Salvia officinalis* L. |
| 19*** | EB-19 | Üzerlik | Wild rue | *---* | *---* |
| 20* | EB-20 | Misk ada çayı | Clary sage | *Salvia sclarea* L. | Not suitable for identification*.* |
| 21 | EB-21 | Gojiberry yaprağı | Goji berry | *Lycium barbarum* L. | *Lycium barbarum* L. |
| 22 | EB-22 | Isırgan | Common nettle | *Urtica dioica* | *Urtica dioica* L. |
| 23 | EB-23 | Aronia | Aronia | *Aronia melanocarpa fructus* | *Aronia melanocarpa* (Michx.) Elliott |
| 24 | EB-24 | Mavi hindiba | Common chicory | *Cichorium intybus* | *Cichorium intybus* L. |
| 25* | EB-25 | Karahindiba | Common dandelion | *Taraxacum officinale* | Not suitable for identification*.* |
| 26 | EB-26 | Aynısefa | Mary's gold | *Calendula officinalis* | *Calendula officinalis* L. |
| 27* | EB-27 | Şahtere otu | Common fumitory | *Fumaria officinalis* | Not suitable for identification*.* |
| 28** | EB-28 | Biberiye | Rosemary | *Rosmarinus officinalis* | *Salvia rosmarinus* Spenn. |
| 29 | EB-29 | Üzüm yaprağı | Grape leaf | *Vitis vinifera* L. | *Vitis vinifera* L. |
| 30* | EB-30 | Aslanpençesi | Lady's mantle | *Alchemilla vulgaris* | *Alchemilla* sp. (not suitable for identification at the species level) |
| 31* | EB-31 | Civanperçemi | Yarrow | *Achillea millefolium* | *Achillea* sp. (not suitable for identification at the species level) |
| 32 | EB-32 | Pelin Otu | Wormwood | *Artemisia absinthium* | *Artemisia absinthium* L. |
| 33 | EB-33 | Alıç yaprağı | Oriental hawthorn | *Crataegus orientalis* | *Crataegus azarolus* var. *aronia* L. |
| 34* | EB-34 | Aslankuyruğu | Motherwort | *Leonurus cardiaca* | Not suitable for identification*.* |
| 35* | EB-35 | Dulavrat otu | Greater burdock | *Arctium lappa* | *Arctium* sp. (not suitable for identification at the species level) |
| 36* | EB-36 | Kuşburnu | Dog rose | *Rosa canina* | *Rosa* sp. (not suitable for identification at the species level) |
| 37 | EB-37 | Ekinezya | Eastern purple coneflower | *Echinacea purpurea* | *Echinacea purpurea* (L.) Moench |
| 38* | EB-38 | Yakı otu | Great willowherb | *Epilobium hirsutum* | *Epilobium* sp. (not suitable for identification at the species level) |
| 39 | EB-39 | Ahududu yaprağı | Raspberry leaf | *Rubus idaeus L.* | *Rubus idaeus* L. |
| 40 | EB-40 | Keçisedefi | Galega | *Galega officinalis* | *Galega officinalis* L. |
| 41 | EB-41 | Kişniş | Coriander | *Coriandrum sativum* | *Coriandrum sativum* L. |
| 42 | EB-42 | Rezene | Fennel | *Foeniculum vulgare* | *Foeniculum vulgare* Mill. |
| 43 | EB-43 | Kapari | Caper | *Capparis ovata* | *Capparis spinosa* var. *canescens* Coss. |
| 44* | EB-44 | Ölmez çiçek (Altın otu) | Immortelle | *Helichrysum arenarium* | *Helichrysum* sp. (not suitable for identification at the species level) |
| 45 | EB-45 | Dut yaprağı | White mulberry | *Morus alba* | *Morus nigra* L. |
| 46** | EB-46 | Fesleğen | Basil | *Ocimum basilicum* | *Ocimum basilicum* L. |
| 47 | EB-47 | Çobançantası | Shepherd's purse | *Capsella bursa-pastoris* | *Capsella bursa-pastoris* Medik. |
| 48 | EB-48 | Maydonoz | Parsley | *Petroselinum crispum* | *Petroselinum crispum* (Mill.) Fuss |
| 49 | EB-49 | Ceviz yaprağı | Walnut | *Juglans regia* | *Juglans regia* L. |
| 50* | EB-50 | Ardıç | Common juniper | *Juniperus communis* | Not suitable for identification*.* |
| 51 | EB-51 | Söğüt yaprağı | White willow | *Salix alba* L. | *Salix babylonica* L. |
| 52 | EB-52 | Ebegümeci | Mallow | *Malva sylvestris* | *Malva sylvestris* L. |
| 53 | EB-53 | Ökse otu | European mistletoe | *Viscum album* | *Viscum album* subsp. *album* L. |
| 54 | EB-54 | Sofralık nane | Spearmint | *Mentha spicata* | *Mentha spica* subsp*. spicata* L. |
| 55 | EB-55 | Enginar yaprağı | Artichoke | *Cynara cardunculus* var. *Scolymus* L. | *Cynara scolymus* L. |
| 56* | EB-56 | Sideritis (Dağ ada çayı) |  | *Salvia sideritis* | *Sideritis* sp. (not suitable for identification at the species level) |
| 57 | EB-57 | Kış Çayı Mix (İstanbul kekiği, kara mürver, ıhlamur, sığırkuyruğu, sinirli ot, kuşburnu) | Winter Tea Mix | *Thymus vulgaris, Sambucus nigra, Tilliae flos, Verbascum asperuloides, Plantago lanceolata, Rosa canina* | *Origanum vulgare* subsp. *hirtum* (Link) Ietswaart*, Sambucus nigra* L.*, Tilia tomentosa* Moench*, Verbascum* sp.*, Plantago lanceolata* L.*, Rosa* sp. |
| 58 | EB-58 | Kış Çayı (Kara mürver, tıbbi adaçayı, ekinezya, papatya, kuşburnu, tıbbi hatmi) | Winter Tea | *Sambucus Nigra, Salvia officinalis, Echinacea purpurea, matricaria recutita l, Rosa canina, Althea officinalis* | *Matricaria chamomilla* var. *recutita* (L.) Fiori*, Sambucus nigra* L.*, Salvia officinalis* L.*, Echinacea purpurea* (L.) Moench*, Rosa* sp.*, Althaea officinalis* L. |
| 59 | EB-59 | Anne Bebek Çayı (Keçisedefi, melisa, papatya, hatmi, rezene) | Mother and Baby Tea | *Galega officinalis, Melissa officinalis, matricaria recutita l, Althea officinalis, Foeniculum vulgare* | *Galega officinalis* L.*, Foeniculum vulgare* Mill.*, Matricaria chamomilla* var. *recutita* (L.) Fiori*, Melissa officinalis* L. |
| 60 | EB-60 | İyi Geceler Çayı (Melisa, gül, sarı kantaron, papatya, kedi otu) | Good Night Tea | *Melissa officinalis, Rosa damascene, Hypericum perforatum, matricaria recutita l, Valeriana officinalis* | *Rosa damascena* Mill.*, Matricaria chamomilla* var. *recutita* (L.) Fiori*, Hypericum perforatum* L.*, Valeriana officinalis* L.*, Melissa officinalis* L. |

*It was excluded from the study because its content was not identified. **Excluded from the study because no dry matter was obtained as a result of lyophilization. *** Inappropriate for ingestion as a tea

Supplementary Table 2

| **Sample No.** | **Quantity (g)** | **Water (mL)** | **Temperature (°C)** |
| --- | --- | --- | --- |
| 1,16, 23^1^, 32, 41^1^ | 1,5 | 150 | 80 |
| 2, 3, 4, 5, 7, 8, 9, 10, 11, 17, 18, 20, 21, 22, 24, 25, 26, 27, 28, 29, 33, 34, 36^1^, 40, 43, 44, 45, 46, 47, 48, 49, 50, 51, 52, 54, 55, 56, 57, 58, 59 | 2 | 200 | 80 |
| 12, 15, 35, 38, 60 | 2 | 150 | 80 |
| 13^2^ | 2 | 150 | 60 |
| 14^3^, 37, 39 | 3 | 200 | 80 |
| 30 | 1 | 200 | 80 |
| 31 | 1,5 | 200 | 80 |
| 53^4^ | 2,5 | 200 | 25 |
| 6, 42^1^ | 5 | 150 | 80 |

^1^Prior to brewing, it was prepared by gently pulverizing in a mortar and pestle. ^2^The mixture was initially agitated in water at ambient temperature for 20 minutes, subsequently heated to 60°C, and finally filtered. ^3^Initially, it was immersed in water at ambient temperature for 120 minutes, subsequently heated to 80°C and filtered. ^4^The substance was immersed in water at ambient temperature for 12 hours in the absence of light and subsequently filtered.

Supplementary Table 3

| Sample | IC_50_ (µg/mL) | Stock Solution | | First dose | | | Second Dose | | | Third Dose | | |
| --- | --- | --- | --- | --- | --- | --- | --- | --- | --- | --- | --- | --- |
|  | A549 | mg | mL | Sample (µL) | PBS (µL) | Final concentration (µg/mL) | Sample (µL) | PBS (µL) | Final concentration (µg/mL) | Sample (µL) | PBS (µL) | Final concentration (µg/mL) |
| 2 | 157.16 ± 1.89 | 1 | 1 | 8 | 942 | 8 | 16 | 934 | 16 | 32 | 918 | 32 |
| 3 | 132.89 ± 1.92 | 1 | 1 | 7 | 943 | 7 | 14 | 936 | 14 | 28 | 932 | 28 |
| 4 | 99.68 ± 0.99 | 1 | 1 | 5 | 945 | 5 | 10 | 940 | 10 | 20 | 930 | 20 |
| 5 | 503.90 ± 2.48 | 1 | 1 | 25 | 925 | 25 | 50 | 900 | 50 | 100 | 850 | 100 |
| 7 | 390.44 ± 1.03 | 0.5 | 1 | 39 | 911 | 20 | 78 | 872 | 40 | 156 | 794 | 80 |
| 9 | 170.40 ± 1.45 | 0.5 | 1 | 17 | 933 | 9 | 34 | 916 | 17 | 68 | 882 | 34 |
| 11 | 312.99 ± 2.18 | 0.5 | 1 | 31 | 919 | 15 | 62 | 888 | 31 | 124 | 826 | 62 |
| 17 | 280.71 ± 1.39 | 1 | 1 | 14 | 936 | 14 | 28 | 922 | 28 | 56 | 894 | 56 |
| 18 | 299.16 ± 1.47 | 1 | 1 | 15 | 935 | 15 | 30 | 920 | 30 | 60 | 890 | 60 |
| 29 | 388.30 ± 2.76 | 1 | 1 | 20 | 930 | 20 | 39 | 911 | 39 | 78 | 872 | 78 |
| 37 | 440.29 ± 1.38 | 0.5 | 3 | 132 | 818 | 22 | 264 | 686 | 44 | 528 | 422 | 88 |
| 54 | 236.17 ± 1.36 | 1 | 1 | 12 | 938 | 12 | 24 | 926 | 24 | 48 | 902 | 48 |

A549: Human lung cancer cell line. PBS: Phosphate-buffered saline

Supplementary Table 4

| Sample | IC_50_ (µg/mL) | Stock Solution | | First dose | | | Second Dose | | | Third Dose | | |
| --- | --- | --- | --- | --- | --- | --- | --- | --- | --- | --- | --- | --- |
|  | A549 | mg | mg | Sample (µL) | PBS (µL) | Final concentration (µg/mL) | Sample (µL) | PBS (µL) | Final concentration (µg/mL) | Sample (µL) | PBS (µL) | Final concentration (µg/mL) |
| 2 | 157.16 ± 1.89 | 1 | 1 | 22 | 942 | 8 | 43 | 934 | 16 | 86 | 918 | 32 |
| 3 | 132.89 ± 1.92 | 1 | 1 | 19 | 943 | 7 | 38 | 936 | 14 | 76 | 932 | 28 |
| 4 | 99.68 ± 0.99 | 1 | 1 | 14 | 945 | 5 | 27 | 940 | 10 | 54 | 930 | 20 |
| 5 | 503.90 ± 2.48 | 1 | 1 | 68 | 925 | 25 | 135 | 900 | 50 | 270 | 850 | 100 |
| 7 | 390.44 ± 1.03 | 0.5 | 1 | 105 | 911 | 20 | 211 | 872 | 40 | 422 | 794 | 80 |
| 9 | 170.40 ± 1.45 | 0.5 | 1 | 46 | 933 | 9 | 92 | 916 | 17 | 184 | 882 | 34 |
| 11 | 312.99 ± 2.18 | 0.5 | 1 | 84 | 919 | 15 | 167 | 888 | 31 | 334 | 826 | 62 |
| 17 | 280.71 ± 1.39 | 1 | 1 | 38 | 936 | 14 | 76 | 922 | 28 | 152 | 894 | 56 |
| 18 | 299.16 ± 1.47 | 1 | 1 | 40 | 935 | 15 | 81 | 920 | 30 | 162 | 890 | 60 |
| 29 | 388.30 ± 2.76 | 1 | 1 | 54 | 930 | 20 | 105 | 911 | 39 | 210 | 872 | 78 |
| 37 | 440.29 ± 1.38 | 0.5 | 3 | 356 | 818 | 22 | 713 | 686 | 44 | 1426 | 422 | 88 |
| 54 | 236.17 ± 1.36 | 1 | 1 | 32 | 938 | 12 | 65 | 926 | 24 | 130 | 902 | 48 |
| A549: Human lung cancer cell line. PBS: Phosphate-buffered saline | | | | | | | | | | | | |
